# Effect of the Rehabilitation Program, ReStOre, on Serum Biomarkers in a Randomized Control Trial of Esophagogastric Cancer Survivors

**DOI:** 10.3389/fonc.2021.669078

**Published:** 2021-09-15

**Authors:** Susan A. Kennedy, Stephanie L. Annett, Margaret R. Dunne, Fiona Boland, Linda M. O’Neill, Emer M. Guinan, Suzanne L. Doyle, Emma K. Foley, Jessie A. Elliott, Conor F. Murphy, Annemarie E. Bennett, Michelle Carey, Daniel Hillary, Tracy Robson, John V. Reynolds, Juliette Hussey, Jacintha O’Sullivan

**Affiliations:** ^1^Trinity St. James's Cancer Institute, Trinity Translational Medicine Institute, Department of Surgery, St. James’s Hospital, Trinity College Dublin, Dublin, Ireland; ^2^School of Pharmacy and Biomolecular Science, Royal College of Surgeons Ireland, Dublin, Ireland; ^3^Data Science Centre, Royal College of Surgeons Ireland, Dublin, Ireland; ^4^Discipline of Physiotherapy, School of Medicine, Trinity College Dublin, Dublin, Ireland; ^5^School of Medicine, Trinity College Dublin, Dublin, Ireland; ^6^School of Biological Sciences, Dublin Institute of Technology, Dublin, Ireland; ^7^School of Mathematics & Statistics, University College Dublin, Dublin, Ireland

**Keywords:** esophagogastric cancer, exercise training, cardiorespiratory fitness, biomarker analysis, multidisciplinary rehabilitation treatment, cancer survivorship, cancer rehabilitation

## Abstract

**Background:**

The Rehabilitation Strategies Following Esophagogastric cancer (ReStOre) randomized control trial demonstrated a significant improvement in cardiorespiratory fitness of esophagogastric cancer survivors. This follow-up, exploratory study analyzed the biological effect of exercise intervention on levels of 55 serum proteins, encompassing mediators of angiogenesis, inflammation, and vascular injury, from participants on the ReStOre trial.

**Methods:**

Patients >6 months disease free from esophagogastric cancer were randomized to usual care or the 12-week ReStOre program (exercise training, dietary counselling, and multidisciplinary education). Serum was collected at baseline (T0), post-intervention (T1), and at 3-month follow up (T2). Serum biomarkers were quantified by enzyme-linked immunosorbent assay (ELISA).

**Results:**

Thirty-seven patients participated in this study; 17 in the control arm and 20 in the intervention arm. Exercise intervention resulted in significant alterations in the level of expression of serum IP-10 (mean difference (MD): 38.02 (95% CI: 0.69 to 75.35)), IL-27 (MD: 249.48 (95% CI: 22.43 to 476.53)), and the vascular injury biomarkers, ICAM-1 (MD: 1.05 (95% CI: 1.07 to 1.66)), and VCAM-1 (MD: 1.51 (95% CI: 1.04 to 2.14)) at T1. A significant increase in eotaxin-3 (MD: 2.59 (95% CI: 0.23 to 4.96)), IL-15 (MD: 0.27 (95% CI: 0 to 0.54)) and decrease in bFGF (MD: 1.62 (95% CI: -2.99 to 0.26)) expression was observed between control and intervention cohorts at T2 (p<0.05).

**Conclusions:**

Exercise intervention significantly altered the expression of a number of serum biomarkers in disease-free patients who had prior treatment for esophagogastric cancer.

**Impact:**

Exercise rehabilitation causes a significant biological effect on serum biomarkers in esophagogastric cancer survivors.

**Clinical Trial Registration:**

ClinicalTrials.gov (NCT03314311).

## Introduction

While the historical advice to cancer patients was rest and avoidance of physical activity, there is now compelling evidence which demonstrates that physical activity is not only safe for cancer survivors but extremely beneficial in improving health related quality of life, reducing levels of anxiety, fatigue, and depression (1). A significant rise in the number of cancer survivors worldwide is expected by 2040, which currently stands at approximately 44 million (1, 2). At the end of 2019, three seminal papers outlining the benefits of physical activity in cancer prevention, recurrence and treatment related side-effects were published following a roundtable review by the American College of Sports Medicine (ACSM) expert panel (1–3). As physical activity can impact cellular processes by altering the endogenous systemic milieu (3), this study examined a range of serum biomarkers in esophagogastric cancer survivors.

Prior to diagnosis, esophagogastric cancer patients may experience dysphagia and eating difficulties which can result in considerable weight loss and fatigue (4, 5). Moreover, chemotherapy, radiation and surgery have a detrimental effect on health related quality of life (HRQOL) and patients can struggle with loss of appetite, difficulty eating, sarcopenia, fatigue, and long standing post-operative weight loss (4, 6–9). Cardiorespiratory fitness is an important index of health and all-cause mortality and remains impaired in esophagogastric cancer survivorship (10, 11). Exercise rehabilitation strategies in esophagogastric cancer must aim to attenuate or avoid excess weight loss in these patients; however, there is a strong rationale to provide rehabilitation programmes for esophagogastric cancer patients.

The multidisciplinary ReStOre (Rehabilitation Strategies following Esophagogastric Cancer) program was developed to incorporate exercise rehabilitation with 1:1 dietary counselling and patient education sessions. A feasibility study of the ReStOre programme found high recruitment rates, adherence, acceptability and a lack of adverse events (12). A further randomized controlled trial (RCT) utilizing the ReStOre protocol demonstrated that structured exercise rehabilitation leads to meaningful improvements in cardiorespiratory fitness in esophagogastric cancer survivors (13). Following the ReStOre intervention participants experienced a clinically meaningful improvement in VO_2_ peak without weight loss occurring (13). The question remains however, whether these rehabilitation programs affect the serum inflammatory profiles of these patients pre, during, and post exercise intervention, and if so, how.

Esophageal cancer is an exemplar model of an inflammation-driven cancer characterized by the overproduction of cytokines and chemokines such as IL-6, IL-8, IL-10, IL-1β, TGF-α, and TGF-β in the tumor microenvironment driving carcinogenesis (14). In addition, inflammation is also associated with symptoms affecting HRQOL, such as fatigue, stress, and cachexia in cancer patients and survivors (15–17). A systematic review concluded that exercise interventions are beneficial in improving HRQOL in cancer survivors (18) and importantly this may be partially due to the ability of physical activity to attenuate inflammation (19). In the ReStOre feasibility study (n=22 control, n=21 intervention) a significant reduction in inflammatory status from baseline to post intervention, characterized by a decrease of 11.25% in IL-8 (p = 0.03) was observed (20). While not significant, there was also an overall pattern towards lower post-intervention inflammatory status (percentage change IL-1β = −5.83%; IL-6 = −3.89%; TNF-α = −10.87%) (20). Numerous studies have analyzed the expression levels of various biomarkers in a variety of oncology settings, mainly breast (21–23), colorectal, and prostate cancer (24, 25). However, to the best of our knowledge, a comprehensive assessment has not been conducted in the esophageal cancer setting. In order to gain a more comprehensive assessment of the impact of physical activity at a biological level, the aim of this exploratory study was to analyze the secretion levels of 55 angiogenic, inflammatory, chemokine, and cytokine mediators in the serum from esophagogastric cancer survivors participating in the ReStOre randomized clinical trial.

## Methods

### Study Design

Study participants who had completed treatment with curative intent for esophageal, esophagogastric junction or gastric cancer were recruited from the Esophageal and Gastric Center, St. James’s Hospital (SJH), Dublin, Ireland as detailed in ‘The Rehabilitation Strategies Following Esophagogastric cancer’ (ReStOre) randomized control trial (1). Ethical approval was obtained [REC Reference: 2016-02 List 5 (6)] and the trial registered on ClinicalTrials.gov (NCT03314311). Briefly, disease-free patients treated for esophagogastric cancer were randomized into either usual care or the 12-week ReStOre program and outcomes measured included cardiorespiratory fitness, health related quality of life (HRQOL) and body composition, as reported previously (13). This current study includes a subset of patients from the previously reported ReStOre trial (13) in which serum samples were available for each of the measured time points [baseline (T0), post 12 week intervention (T1), and 24 week follow up (T2)]. The primary outcome measure of this study was blood biomarker analysis following exercise intervention.

### Rehabilitation Program

The ReStOre program is a 12-week rehabilitation program consisting of supervised and home-based exercise, dietary counselling, and multidisciplinary counselling, as previously described (1). Briefly, aerobic exercise training commenced at a low intensity (30 - 40% heart rate reserve) and progressed weekly to a moderate intensity (45% - 60% heart rate reserve). Resistance exercise commenced at 2 sets of 12 repetition maximum and progressed to 6 sets of 17 repetition maximum. Adherence was monitored using polar heart rate monitors (Polar FT7, China) and exercise diaries. Participants received 1:1 dietary counselling from a registered dietitian. Dietary intake and gastrointestinal symptoms were assessed using a 24-hour dietary recall, semi-structured dietary interviewing, and validated scales of gastrointestinal symptoms. Participants attended seven group education sessions by the multidisciplinary team including a surgeon, dietitian, physiotherapist, occupational therapist and a psychotherapist specialized in mindfulness.

### Measures of Physical Performance and Body Composition

Measures were assessed at baseline (T0), immediately post 12-week intervention (T1), and at 3-month follow-up (T2, week 24) as outlined in the ReStOre randomized control trial (1). Cardiorespiratory fitness was determined using a maximal exercise test [cardiopulmonary exercise test (CPET)]. Body composition (anthropometric measurements and bioimpedance analysis), accelerometer measures of physical activity levels, and health related quality of life (HRQOL) questionnaires were recorded.

### Blood Sampling and Biomarker Analysis

Non-fasting venous blood samples were collected from 37/43 ReStOre trial participants (17/22 control and 20/21 intervention, prior to exercise testing at baseline (T0), immediately post-intervention (T1), and at 3-month follow-up (T2). Following 30 min upright rest, samples were centrifuged at 2500 rpm for 10 min at 4°C and serum was cryopreserved at -80°C until analyzed. The V-PLEX Human Biomarker 54-Plex Kit (Meso-Scale Discovery) was used according to manufacturer’s instructions. The 54-plex kit is composed of 7 individual multiplex ELISA assays. The human angiogenesis panel contained: VEGF-A, VEGF-C, VEGF-D, Tie-2, Flt-1, PlGF, and bFGF. The human chemokine panel contained: Eotaxin, MIP-1β, Eotaxin-3, TARC, IP-10, MIP-1α, IL-8 (high abundance), MCP-1, MDC, and MCP-4. The human cytokine panel 1 contained: GM-CSF, IL-1α IL-5, IL-7, IL-12/IL-23p40, IL-15, IL-16, and TNF-β. The human cytokine panel 2 contained: IL-17A/F, IL-17B, IL-17C, IL-17D, IL-1RA, IL-3, IL-9, and TSLP. The human Th17 panel contained: IL-17A, IL-21, IL-22, IL-23, IL-27, IL-31, and MIP-3α. The human pro-inflammatory panel contained: IFN-γ, IL-1β, IL-2, IL-4, IL-6, IL-8, IL-10, IL-12p70, IL-13, and TNF-α. The human vascular injury panel contained SAA, CRP, VCAM-1, and ICAM-1. All 54-plex assays were conducted as per manufacturer’s recommendations, with an alternative protocol of overnight supernatant incubation being used all assays except Vascular Injury and Angiogenesis, which were run in a single day. All results were reported in pg/ml. The anti-angiogenic protein FK506 binding protein like (FKBPL) was analyzed by ELISA (Cloud Clone, USA), as per manufacturer’s instructions. Serum samples were diluted two-fold using standard diluent and loaded as duplicates and FKBPL concentration was calculated using a 4-parameter fit standard curve.

### Statistical Analysis

Statistical analyses were performed using Stata v14 (Ref: StataCorp. 2015. Stata Statistical Software: Release 14. College Station, TX: StataCorp LP) or GraphPad Prism 8.0. Some observations were identified (from boxplots and summary statistics) as outliers and were removed prior to analysis. The exact number of values included in each summary measure and analysis are included in the tables. Descriptive statistics are presented as mean and standard deviation (SD) for continuous variables and frequency (percentage) for categorical data. Initially, associations between baseline serum biomarker and clinical measurements were assessed using Spearman’s rho correlation. Effect sizes were interpreted as 0.2 to 0.5 (weak), 0.51 to 0.80 (moderate), and more than 0.80 strong effect (26). Raw data, was visualized as dot plots, generated in GraphPad Prism (version 8.4.1). Data at time 1 (T1) and time 2 (T2) were analyzed using linear regression models, adjusting for baseline values (T0). When assumptions of linear regression were violated, data were transformed using a log transformation. P-values ≤0.05 were considered statistically significant. There was no adjustment for multiple comparisons.

## Results

Thirty-seven patients participated in this study; 17 in the control arm and 20 in the intervention arm. The mean age of participants was 63 years (SD: 10.1) in the control cohort, and 67 years (SD: 7.7) in the intervention cohort. Mean time post-surgery was in the control group was 37.6 (SD: 19.3) months and 22.6 (SD: 15) months in the intervention group. The majority of patients were diagnosed with adenocarcinoma and tumors were located in the esophagogastric junction (**Table 1**). In the control group, mean baseline BMI was 25.86 kg/m^2^ (SD: 5.11) with a majority (n=10) classed as having a healthy weight BMI, four classed as having an overweight BMI (25-30 kg/m^2^), and three as having an obese BMI (>30 kg/m^2^) (**Table 1**). In the intervention group, mean baseline BMI was 25.44 kg/m^2^ (SD: 3.95) with the majority classed as having a healthy weight BMI (n = 10), six classed as having an overweight BMI (25 -30 kg/m^2^), and four as having an obese BMI (>30 kg/m^2^). No participants were classed as having an underweight BMI (<18.5 kg/m^2^) (**Table 1**). At baseline (T0), the control group had a higher VO_2_ peak (mean 22.85, SD: 4.54 ml/min/kg) compared to the intervention group (mean 18.76, SD: 4.17 ml/min/kg) (**Table 1**). Compared to population norms (27), twenty-two participants had very poor cardiorespiratory fitness, eleven had poor fitness and four had a fair level of fitness at baseline (T0) (**Table 1**).

**Table 1:** Patient Demographics.

PARAMETER	Control (n = 17)*	Intervention (n = 20)*
Gender (M/F)	13M:4F	17M:3F
Mean age (years)	63 (SD: 10.1)	67 (SD: 7.7)
Mean BMI (kg/m^2^)	25.86 (SD: 5.11)	25.44 (SD: 3.95)
BMI category	10 Normal weight (59 %)	10 Normal weight (50 %)
	4 Overweight (24 %)	6 Overweight (30 %)
	2 Obesity Class I (12 %)	4 Obesity class I (20 %)
	1 Obesity Class II (6 %)	
Mean time post-surgery (months)	37.6 (SD: 19.3) months	22.6 (SD: 15) months
Tumor histology:		
Adenocarcinoma	14 (82.4%)	14 (70%)
Squamous cell carcinoma	2 (11.8%)	5 (25%)
Associated Barrett’s	1 (5.9%)	1 (5%)
Tumor location:		
Esophagus	3 (17.6%)	8 (40%)
Esophagogastric junction	13 (76,5%)	11 (55%)
Stomach	1 (5.9%)	1 (5%)
Surgical procedure	2 Stage Oesophagectomy (6)	2 Stage Oesophagectomy (9)
	3 Stage Oesophagectomy (2)	3 Stage Oesophagectomy (2)
	Oesophago-Gastrectomy (2)	Sub-total Gastrectomy (1)
	Sub-total Gastrectomy (1)	Total Gastrectomy (1)
	Total Gastrectomy (3)	Transhiatal Oesophagectomy (7)
	Transhiatal Oesophagectomy (3)	
Tumor node metastasis (TNM) staging	T1N0M0 (3), T2N0M0 (1), T2N1M0 (1), T2N2M0 (1), T3N0M0 (4), T3N1M0 (2), T3N2M0 (4), TisN0M0 (1)	T1N0M0 (6), T1N1M0 (1), T2N0M0 (2), T3N0M0 (2), T3N1M0 (6), T3N2M0 (1), TisN0M0 (2)
Mean baseline VO_2_ peak, ml.min^-1^.kg^-1^	22.85 (SD: 4.54)	18.76 (SD:4.17)
Baseline fitness category		
Poor/Fair	9 (47.06%)	6 (70%)
Very poor	8 (52.94%)	14 (30%)

*Data are presented as the mean and standard deviation (SD) for continuous variable and as frequency (percentage) for categorical variables.

### Correlation Analysis Between Baseline Clinical Measurement and Serum Biomarkers

Some evidence of correlation was observed between serum biomarkers and BMI, namely IL-12/IL-23p40 and TNF-β. A moderate positive correlation was observed at baseline (T0) between BMI and the pro-inflammatory cytokines IL-12/IL-23p40 (rho=0.51, p=0.001) and TNF-β (rho=0.53, p=0.002), while a weak correlation was observed with IL-17A (rho=0.335, p=0.043) **Table 2** and **Supplementary Table 1**. IL-1 receptor antagonist (IL-1RA) (rho=0.39, p=0.02) and IL-17A (rho=0.34, p=0.04) were weakly positively correlated to BMI (**Table 2** and **Supplementary Table 1**). The angiogenic factor, VEGF, also weakly correlated to BMI (rho=0.34, p=0.04) (**Table 2** and **Supplementary Table 1**). The 6-minute walk test (6-MWT) is a sub-maximal exercise test used to assess aerobic capacity and endurance (28). A weak positive correlation between 6-MWT and the anti-inflammatory cytokine IL-10 (rho=0.37, p=0.03) (**Table 2** and **Supplementary Table 2**). Furthermore, a weak, negative correlation was observed with a greater 6-MWT distance and the pro-inflammatory cytokines IL-17A (rho= -0.34, p=0.04), IL-17D (rho=-0.33, p=0.05) and MIP-3α (rho=-0.37, p=0.02) (**Table 2** and **Supplementary Table 2**). No further evidence of correlations were observed with 6-MWT.

**Table 2:** Correlation analysis between clinical measurements and serum biomarkers.

Clinical measurement	Serum biomarker	rho	95% confidence interval	P value (two tailed)
**Body Mass Index**	IL-12/IL-23p40	0.51	0.21 to 0.72	<0.01
	IL-17A	0.33	<0.01 to 0.60	0.04
	IL-1RA	0.39	0.07 to 0.64	0.02
	TNF-β	0.53	0.21 to 0.74	<0.01
	VEGF	0.34	0.01 to 0.60	0.04
**6-Minute Walk Test**	IL-10	0.37	0.04 to 0.62	0.03
	IL-17A	-0.34	-0.61 to -0.01	0.04
	IL-17D	-0.33	-0.60 to 0.01	0.05
	MIP-3α	-0.37	-0.62 to -0.04	0.02
**Anaerobic Threshold**	IFN-γ	-0.34	-0.60 to -0.01	0.04
	IL-17A	-0.35	-0.61 to -0.02	0.03
	IL-17B	-0.43	-0.67 to -0.12	0.01
	IP-10	-0.37	-0.62 to -0.04	0.02
**VO_2_ Max**	IL-17A	-0.35	-0.61 to -0.02	0.03
	VEGF-D	-0.37	-0.63 to -0.04	0.02

The anaerobic threshold (AT), a measure of endurance which calculates the exertion level between aerobic and anaerobic training (3) showed a weak negative correlation with pro-inflammatory cytokines and chemokines including IFN-γ (rho=-0.34, p=0.04), IL-17A (rho=-0.35, p=0.03), IL-17B (rho=-0.43, p=0.01) and IP-10 (rho=-0.37, p=0.02) (**Table 2** and **Supplementary Table 3**). VO_2_max is the maximum rate of oxygen consumption measured during incremental exercise and it is reflective of cardiorespiratory fitness (4). A larger VO_2_max was weakly negatively associated with the pro-inflammatory cytokine IL-17A (rho=-0.35, p=0.03) and VEGF-D (rho=-0.37, p=0.02); a mitogen important for both angiogenesis and lymphangiogenesis (**Table 2** and **Supplementary Table 4**) (5). There was no correlation observed between fatigue score and any of the 55 serum biomarkers measured (**Supplementary Table 5**).

### Exercise Intervention Increases the Expression of ICAM-1 and VCAM-1

**Figure 1:**
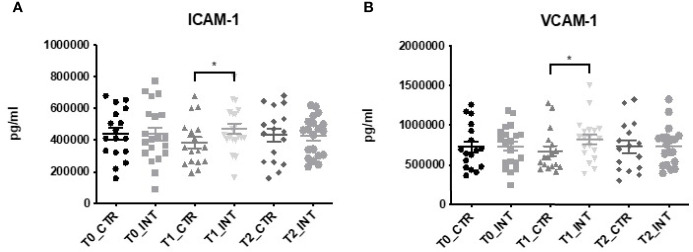
Multiplex ELISA analysis of Vascular injury panel. Data at immediately post 12-week intervention (Time 1; T1) and at 3-month follow-up (Time 2; T2) was analyzed using linear regression models, adjusting for baseline values (T0). A significant increase in serum **(A)** ICAM-1 and **(B)** VCAM-1 expression was observed at T1 (*p ≤ 0.05).

Linear regression analysis of the vascular injury panel which included ICAM-1 (**Figure 1A**) and VCAM-1 (**Figure 1B**) demonstrated significant differences between the control and intervention groups at T1 but not T2 (**Supplementary Table 6**). There was no evidence of significant differences in expression levels of serum amyloid A (SAA) and CRP observed at T1 or T2 (**Supplementary Table 6**). The expression level of ICAM-1 was significantly higher in the intervention compared to the control (ratio of means: 1.05 (95% confidence interval (CI): 1.07 to 1.66); p=0.02) at T1 (**Figure 1A**). Similarly, a significantly higher increase in VCAM-1 expression was observed in the intervention cohort compared to the control cohort [ratio of means: 1.51 (95% CI: 1.04 to 2.14); p=0.02] at T1 (**Figure 1B**).

### IP-10 and Eotaxin-3 Expression Increases Following Exercise Intervention

**Figure 2:**
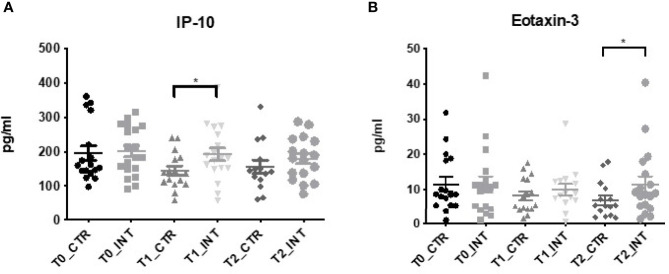
Exercise intervention increases IP-10 and Eotaxin–3 expression. Data at immediately post 12-week intervention (Time 1; T1) and at 3-month follow-up (Time 2; T2) was analyzed using linear regression models, adjusting for baseline values (T0). A significant increase in serum **(A)** IP-10 expression was observed at T1 between control and intervention cohorts while a significant increase in **(B)** Eotaxin-3 was observed at T2 (*p ≤ 0.05).

Of the panel of chemokine markers assessed, a significant increase in IP-10 expression was detected in the intervention group compared to the control group at T1 [mean difference (MD) 38.02 pg/ml (95% CI: 0.69 to 75.35); p=0.05] (**Figure 2A** and **Supplementary Table 6**). No significant difference in expression was observed at T2 (p=0.61) (**Figure 2A** and **Supplementary Table 6**). Furthermore, there was significant increase in eotaxin-3 in the intervention compared to the control at T2 (MD: 2.59 (95% CI: 0.23 to 4.96), p=0.03) (**Figure 2B** and **Supplementary Table 6**) at T2. No significant difference in expression levels was observed for the remaining chemokine panel members eotaxin, MIP-1β, TARC, IP-10, MIP-1α, IL-8, MCP-1, MDC and MCP-4 (**Supplementary Table 6**).

### IL-27 Expression Increases Following Exercise Intervention

**Figure 3:**
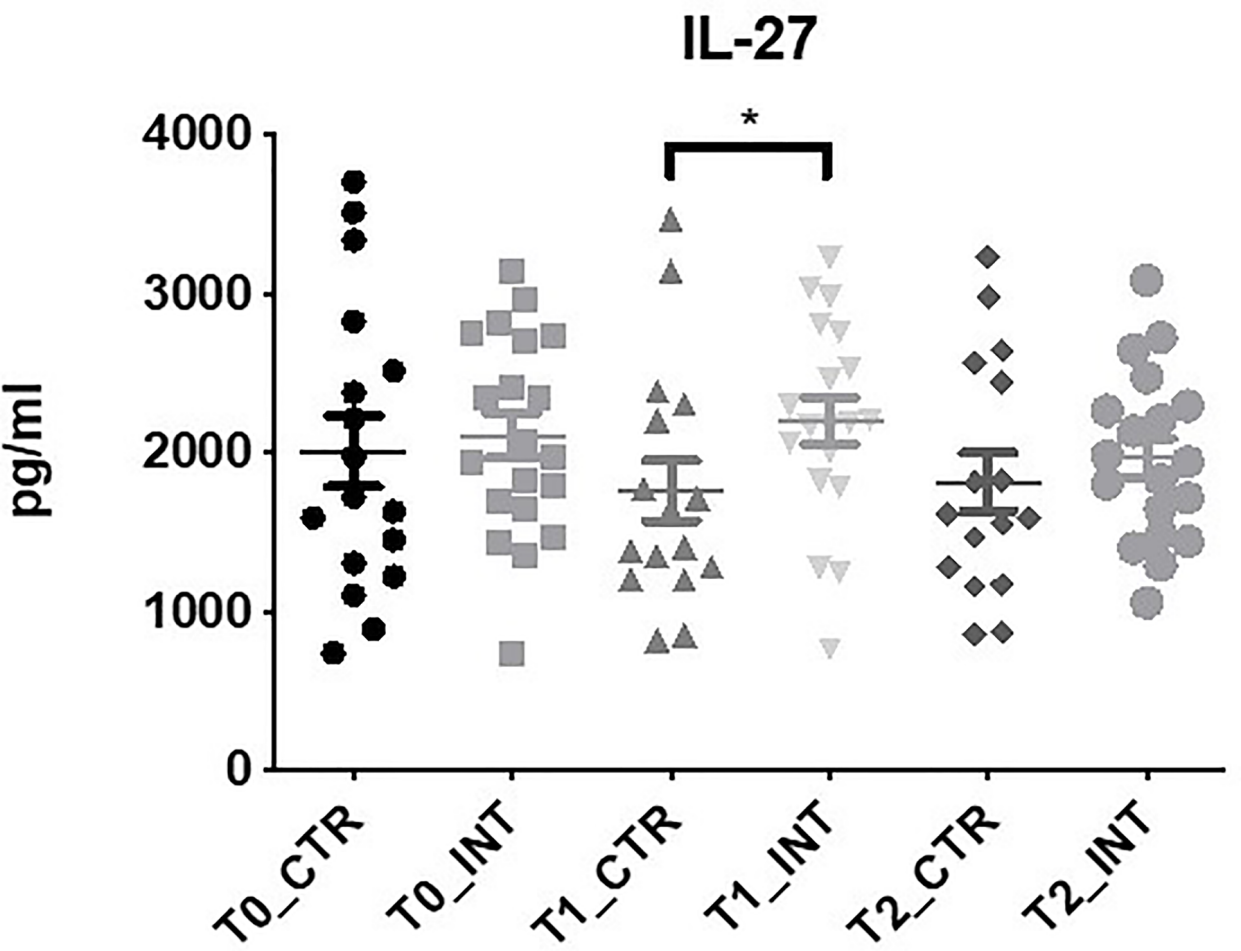
Exercise intervention increases IL-27 expression at T1. Data at immediately post 12-week intervention (Time 1; T1) and at 3-month follow-up (Time 2; T2) was analyzed using linear regression models, adjusting for baseline values (T0). A significant increase in serum IL-27 expression was observed at T1 between control and intervention cohorts (*p ≤ 0.05).

IL-27 expression was significantly increased in the intervention compared to the control cohort at T1 (MD: 249.48 (95% CI 22.43 to 476.53), p=0.03), at T1 p=0.03 (**Figure 3** and **Supplementary Table 6**). There was no significant difference found between control and intervention cohorts for IL-17A, IL-22 and MIP-3α at T1 or T2 (**Supplementary Table 6**). IL-21 and IL-23 were below the level of detection. Statistical analysis could not be conducted for IL-31 as expression was only detected in a small number of patients.

### bFGF Expression Decreases Following Exercise Intervention

**Figure 4:**
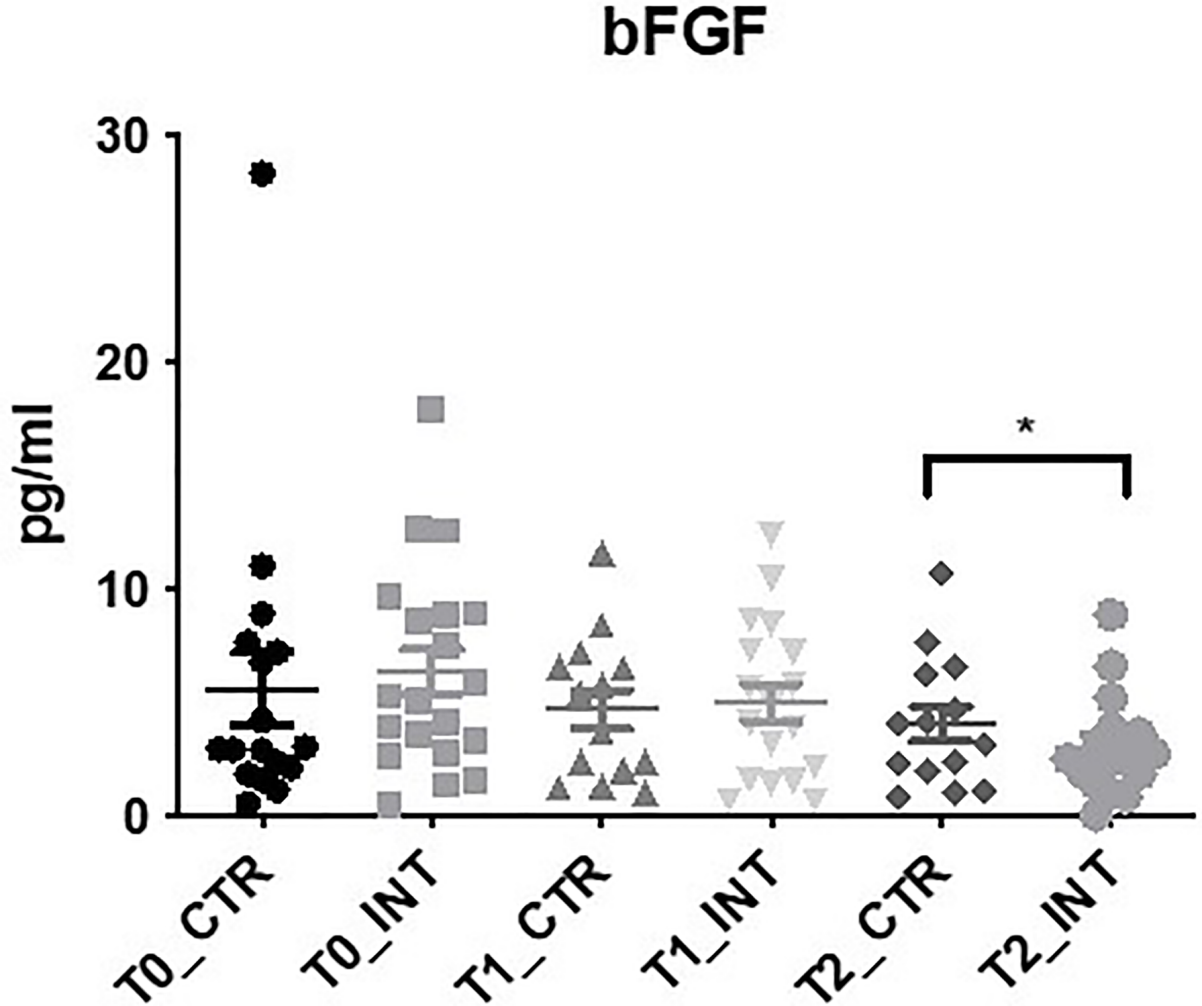
Exercise intervention decreases bFGF expression at T2. Data at immediately post 12-week intervention (Time 1; T1) and at 3-month follow-up (Time 2; T2) was analyzed using linear regression models, adjusting for baseline values (T0). A significant decrease in serum bFGF expression was observed at T2 between control and intervention cohorts (*p ≤ 0.05).

In the panel of angiogenesis markers bFGF expression was significantly decreased at 3-month follow-up (T2) in the intervention cohort compared to the control cohort (MD: -1.62 (95% CI -2.99 to -0.26), p=0.02), (**Figure 4** and **Supplementary Table 6**). No significant difference in bFGF expression was observed at T1 (p=0.08) (**Figure 4** and **Supplementary Table 6**). In addition, there was no significant difference found between control and intervention groups for angio VEGF, Flt-1, PlGF, VEGF, VEGF-C, VEGF-D, TIE-2, and FKBPL (**Supplementary Table 6**).

### IL-15 Expression Increases Following Exercise Intervention

**Figure 5:**
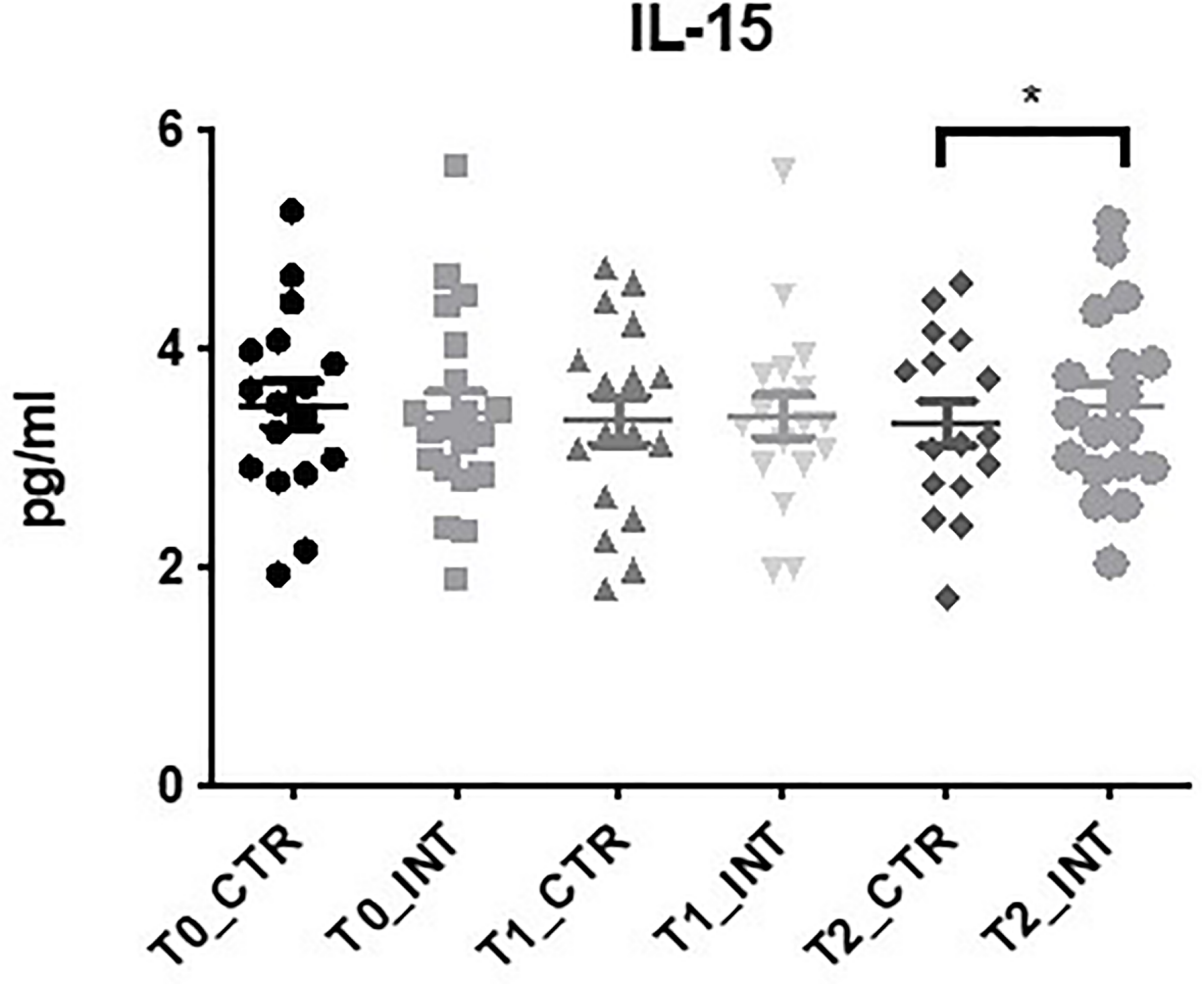
Exercise intervention increases IL-15 expression at T2. Data at immediately post 12-week intervention (Time 1; T1) and at 3-month follow-up (Time 2; T2) was analyzed using linear regression models, adjusting for baseline values (T0). A significant increase in serum IL-15 expression was observed at T2 between control and intervention cohorts (*p ≤ 0.05).

Eighteen cytokine makers were analyzed in this study. The expression level of IL-15 was the only cytokine found to be significantly altered following exercise intervention (**Figure 5** and **Supplementary Table 6**). At T2, a significant increase (p=0.05) in expression was observed in the intervention cohort compared to the control cohort (MD: 0.27 (95% CI: <0.01 to 0.54), p=0.05). No significant change in the expression levels of the remaining cytokine markers were observed (**Supplementary Table 6**), or cytokine panel 2 (**Supplementary Table 6**). The expression levels of IL-3 and IL-17C were below the level of detection. Statistical analysis could not be conducted for GMCSF, IL-1α, IL-17A/F, and IL-31 as expression was only detected in a small number of patients.

## Discussion

The benefits of physical activity are multi-factorial, from reducing cancer incidence, lowering the risk of recurrence and improving patient quality of life (29, 30). The ReStOre RCT has demonstrated that structured exercise rehabilitation resulted in improved confidence, physical, social and mental wellbeing of participants (13). This follow-up study analyzed the effect of physical activity on 55 serum biomarkers and enabled a more comprehensive assessment of angiogenic, inflammatory, cytokine and chemokine expression levels which may be altered following exercise intervention. Published research on the benefits of exercise in the cancer population has been dominated by breast cancer studies (1, 31, 32). To our knowledge, this is the largest screening analysis on the effect of exercise on serum inflammatory biomarkers in the esophageal cancer survivorship setting. In this study we have demonstrated that physical activity alters the secretion of a number of markers including eotaxin-3, IP-10, bFGF, IL-27, ICAM-1, and VCAM-1. Additional studies ongoing in the esophageal cancer setting include the PERFECT study, which included participants with esophageal cancer after surgery with curative intent in a 12-week exercise intervention. Secondary outcome measures of this study include blood marker analysis which at the time of writing have yet to be published (33). During the period between the intervention finishing (T1) and the 12 week post intervention follow up (T2), participants did not attend supervised exercise sessions, but a strong focus on self-management was instilled in participants to promote adherence to the exercise program in an unsupervised basis. Adherence to exercise was reflected in cardiorespiratory fitness levels measured by VO_2_ peak which were consistently higher in the intervention cohort compared to control at T1 and T2 (13).

A meta-analysis by Meneses-Echávez et al., has shown that circulating IL-6, which has been also associated with symptoms of fatigue, IL-8 and TNF-α were reduced in breast cancer survivors following regular exercise (23). While no significant alteration in the expression levels of IL-6, IL-8, TNF-α, or any of the pro-inflammatory’ cytokines were observed in this study (**Supplementary Table 6**), this could possibly be due to the duration of the exercise intervention. The mean exercise intervention in the meta-analysis conducted by Meneses-Echávez et al., was performed for 19 ± 13 weeks (23), indicating a longer intervention may have been required to observe significant changes in expression levels. Also, possibly the secretion of these inflammatory mediators may display cyclic profiles with time. Interestingly, our 12-week exercise intervention did significantly alter the expression levels of soluble intracellular adhesion molecular (ICAM-1) and soluble vascular adhesion molecule (VCAM-1), members of the vascular injury panel at T1 compared to controls. Both soluble ICAM-1 and VCAM-1 are reported to be increased following exercise and circulating levels of cells are important in skeletal muscle remodeling (34–36). Similarly, we also found the exercise intervention group had elevated ICAM-1 and VCAM-1 at T1 compared to controls (**Figure 1**). Other members of the vascular injury panel, CRP and SAA, demonstrated no significant difference in serum expression levels following exercise intervention. However, other clinical studies have shown that a longer intervention time (>16 weeks) is required to detect changes in the systemic levels of pro-inflammatory makers such as CRP, TNF-α and IL-6 which are shown to increase following physical inactivity or sedentary behavior (29, 37).

Basic fibroblast growth factor (bFGF) is a polypeptide growth factor which drives tumorigenesis through both the proliferation of tumor cells and the promotion of angiogenesis (38). Indeed, in esophageal cancer, high bFGF expression was associated with tumor progression and significantly correlated with depth of invasion, lymph-node metastasis, TNM stage and micro vessel density (39). A significant decrease in bFGF levels was identified at T2 (p=0.02), 24-weeks after commencing the exercise intervention, in the intervention group compared to the control group (**Figure 4**). To the best of our knowledge, this is the first study to investigate bFGF levels in cancer survivors following exercise intervention. Increased mRNA bFGF expression was previously observed in rat skeletal muscle following a single exercise bout (40), while serum bFGF levels significantly increased in overweight Japanese men 6-months after commencing an exercise intervention (41) Notably, serum bFGF levels were inversely correlated with both a reduction in BMI and improved exercise capacity (41). A key difference with the ReStOre RCT is that participants were monitored to ensure their BMI did not decrease, as unintentional weight loss is problematic in esophagogastric cancer survivors due to long-term nutritional challenges. Several studies have noted elevated levels of bFGF in inflammatory bowel conditions (42–44). In addition, inflammatory stimuli have been shown to increase bFGF levels (45, 46). One possible explanation for the decrease in bFGF at 3-month follow up (T2) is a decrease in systematic inflammation in the intervention cohort. A longer intervention and follow-up may be required to detect changes in other pro-inflammatory stimuli measured within the study. Moreover, bFGF levels are significantly upregulated in esophageal and gastroesophageal junction adenocarcinomas and Barrett’s esophagus; a precursor lesion in this cohort (47). Therefore, it is very encouraging to observe a decrease of serum bFGF in the intervention cohort.

Interferon gamma-induced protein (IP-10), also known as, CXCL10, is an immunomodulatory cytokine associated with lymphocytic infiltrate to the tumor site. High levels have been associated with poor survival in patients in breast (48), pancreatic (49), and esophageal (50) cancer. We found that baseline levels of IP-10 and aerobic threshold were negatively correlated (**Supplementary Table 3**). Additionally, a significant increase in IP-10 expression was observed in the intervention cohort at T1, following completion of the 12-week program, but not at the second 3-month follow up (T2), despite there being an enhancement in cardiovascular fitness at both time points (**Figure 2**). In contrast to our findings, a large RCT of healthy adults (n=413) reported that 8 weeks of exercise training was associated with a decrease in IP-10 and a 12-week exercise program in obese adolescents also observed a decrease in IP-10 serum concentration (51, 52). However, it is important to note that while a decrease in serum IP-10 was observed, both studies were conducted in a previously healthy or younger cohort with higher levels of elevated cardio respiratory fitness at enrolment in comparison to participants of the ReStOre RCT. It is well-reported that exercise may increase acute phase proteins and inflammatory markers, as observed in this study (**Figure 1**). Indeed inflammatory cytokines are released from skeletal muscle, resulting in enhanced plasma levels related to exercise duration, intensity and muscle mass (53, 54). However, after regular exercise, fewer inflammatory markers are released and long term adaption results in an increase release of anti-inflammatory substances (54). IP-10 (aka CXCL10) is an exercise controlled myokine (55) and therefore it is not surprising that changes in expression were observed in the intervention group. Further studies in a diverse range of subjects are required to precisely understand the role of IP-10 in physical activity.

IL-27 is a member of the IL-6/IL-27 family produced by antigen presenting cells with both pro and anti-tumorigenic properties (56, 57). We demonstrated an increase in serum IL-27 expression at T1 following exercise intervention (**Figure 3**). High serum IL-27 level is associated with cancer presence and lymph node metastases in gastroesophageal cancer (58). Little is known about how exercise impacts IL-27 in isolation, however, as a member of the subfamily of cytokines which signals through glycoprotein-130, it could play a role in the regulation of skeletal muscle remodeling (59, 60). Eotaxin-3, also known as CCL26, belongs to the CC cytokine family and it acts as a chemoattractant for eosinophils and basophils in allergic disorders (61). No studies to date have investigated the role of eotaxin-3 in esophageal cancer, however in colon cancer, eotaxin-3 induced tumor associated macrophages by binding to the CCR3 receptor and enhancing the invasiveness of tumor cells (62). Similarly, in prostate cancer mesenchymal stem cells modulate of the invasive potential of prostate cancer cells *via* the Eotaxin-3/CCR3 axis (63). We noted a significant increase in eotaxin-3 levels in the intervention group compared to the control group at T2 (**Figure 2** and **Supplementary Table 6**). To our knowledge, no other study has investigated the effect of exercise intervention on eotaxin-3 levels, however the HEPAFIT RCT currently underway aims to access the effect of an exercise programme on overweight/obese adolescents and the study protocol states that eotaxin-3 will be measured (64).

Of the eleven cytokines analyzed in this study, IL-15 was the only cytokine family member observed to be significant. IL-15 is a member of the 4a-helix bundle cytokine, a myokine which is released by the muscle following exercise (65). The expression of IL-15 is high in skeletal muscle (66) and variability in plasma IL-15 expression has been shown following resistance training. In a 10-week acute resistance training program, Riechman et al., demonstrated increased plasma IL-15 expression in 127 participants post-training (67), however, this was not observed in a study by Nielson et al., which analyzed IL-15 mRNA, protein, and plasma expression levels in 8 healthy male subjects following heavy resistance training. While mRNA IL-15 expression levels increased in skeletal muscle, there was no significant difference in plasma IL-15 levels following resistance training (65). Interestingly, in this study we did not observe any significant changes in serum IL-15 expression immediately following the 12-week supervised exercise session (T1), however mean expression levels remained constant from T1 to T2 in the intervention cohort while a decrease of IL-15 expression was observed in the T2 control cohort leading to significance (**Figure 5** and **Supplementary Table 6**).

The broad screen of angiogenic, inflammatory, chemokine and cytokine panels analyzed in this study has enabled us to assess serum markers in which (i) expression levels were undetectable, (ii) expression levels were identified in only a small number of patient samples and therefore could not be analyzed statistically, and (iii) expression levels were detected and warrant further investigation in a larger patient cohort. Of the 55 markers analyzed in our study, statistically significant results were seen for only 7. This could be due to the sample size and levels of biological heterogeneity observed in the control (n=17) and intervention (n=20) groups. One of the limitations of this study is the relatively small sample size given the number of tests and associations explored. The study was not powered to test for these associations, hence the exploratory nature of this study. Additionally, no adjustment for multiple comparisons was used. Hence, all results should be interpreted with caution. Furthermore, as stated previously, a longer exercise intervention (>12-weeks) may have yielded more significant variation in systemic markers (29, 68). While a longer exercise intervention period may be required to demonstrate changes in inflammatory markers, a single exercise session has been shown to reduce side effects of chemotherapy and reduce nausea in a cohort of women undergoing treatment for breast cancer (22). With the multitude of recent evidence, the benefits of exercise as an added therapeutic intervention in cancer treatment are clear, particularly in aiding and relieving treatment related side effects such as fatigue, nausea, and vomiting (21, 22). Of equal importance is the benefit of exercise to cancer survivors who experience deficits in cardiorespiratory fitness which impact daily activities. While there is insufficient literature at present on personalized exercise prescriptions, a future goal of exercise oncology is tailoring exercise programs specific to the individual patient’s cancer and their treatment type (1). The ReStOre RCT which combines both aerobic and resistance training, previously demonstrated the positive impacts of a 12-week supervised exercise intervention on participant physical, mental, and social well-being (13). In this study we demonstrate the biological effect of exercise intervention in significantly modulating a number of serum biomarkers involved in inflammation and metastasis. The ReStOre II clinical trial is currently underway and is recruiting a total of 120 patients who have had curative treatment for upper gastrointestinal (UGI) and hepatopancreaticobiliary (HPB) cancers (69). A secondary outcome of the trial is to establish an UGI cancer survivorship biobank for collaborative translational research studies (69) and therefore the results of this study will be validated in this larger cohort.

## Data Availability Statement

The original contributions presented in the study are included in the article/**Supplementary Material**. Further inquiries can be directed to the corresponding author.

## Ethics Statement

The studies involving human participants were reviewed and approved by Esophageal and Gastric Center, St. James’s Hospital (SJH), Dublin, Ireland. The patients/participants provided their written informed consent to participate in this study.

## Author Contributions

Conception and design: JO’S, JH, and JR. Development of methodology: LO’N, EG, SD, EF, JE, CM, and AB. Acquisition of data: SK, SA, EF and MD. Analysis and interpretation of data: SK, SA, FB, MC, and DH. Writing, review, and/or revision of the manuscript: SK, SA, and JO’S. Study supervision: JO’S, JH, JR, and TR. All authors contributed to the article and approved the submitted version.

## Funding

SK was funded by Science Foundation Ireland 17/TIDA/5053. SA and TR were awarded funding through the National Children’s Research Centre and the Children’s Medical & Research Foundation, Crumlin, Ireland, grant code (C/18/9). TR was also funded through the Science Foundation Ireland strategic partnership programme, Precision Oncology Ireland, grant code (18/SPP/352). LON, EG, SD, JH, and JOS were awarded funding by the Health Research Board Ireland (The ReStOre trial, grant number HRA-POR-2014-535).

## Conflict of Interest

The authors declare that the research was conducted in the absence of any commercial or financial relationships that could be construed as a potential conflict of interest.

## Publisher’s Note

All claims expressed in this article are solely those of the authors and do not necessarily represent those of their affiliated organizations, or those of the publisher, the editors and the reviewers. Any product that may be evaluated in this article, or claim that may be made by its manufacturer, is not guaranteed or endorsed by the publisher.
