# Supplementary figures and images for: Effect of the Rehabilitation Program, ReStOre, on Serum Biomarkers in a Randomized Control Trial of Esophagogastric Cancer Survivors
Source: Front Oncol. 2021 Sep 15;11:669078. doi: 10.3389/fonc.2021.669078 (PMC8479183; doi:10.3389/fonc.2021.669078)

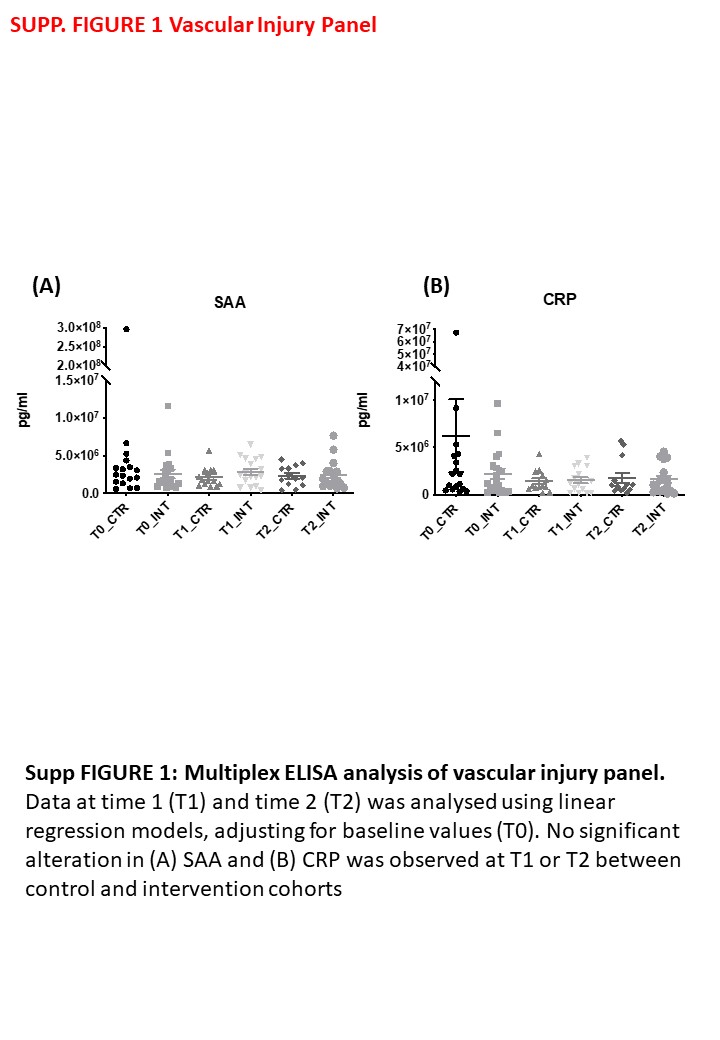

Supplement: Supplementary file 1 [file Image_1.jpeg]

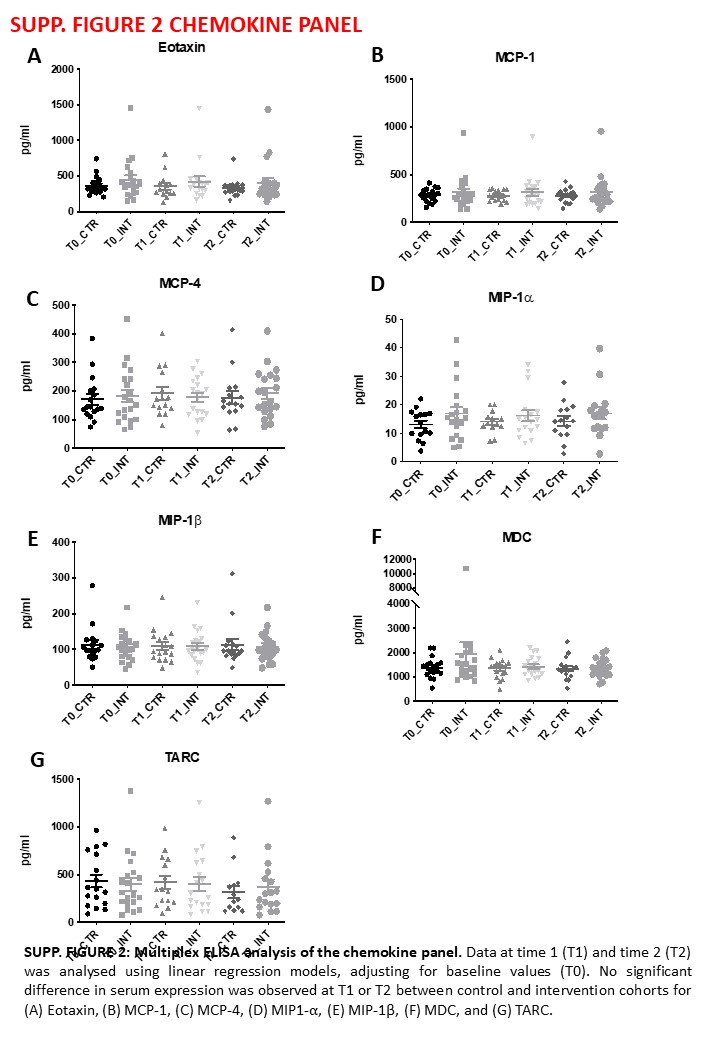

Supplement: Supplementary file 2 [file Image_2.jpeg]

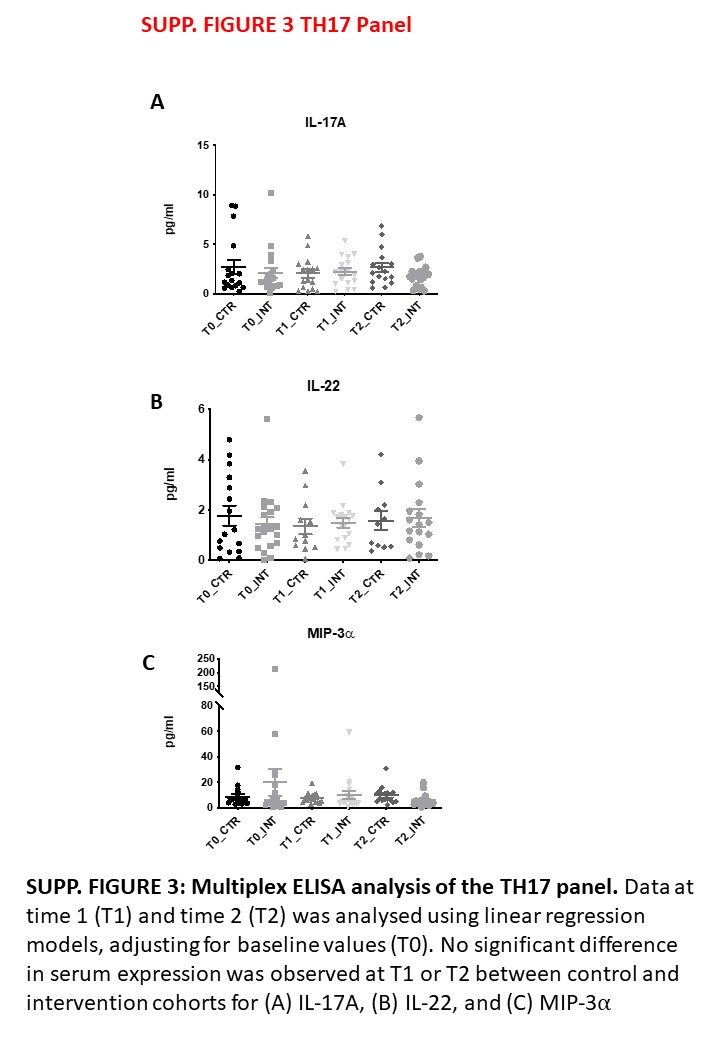

Supplement: Supplementary file 3 [file Image_3.jpeg]

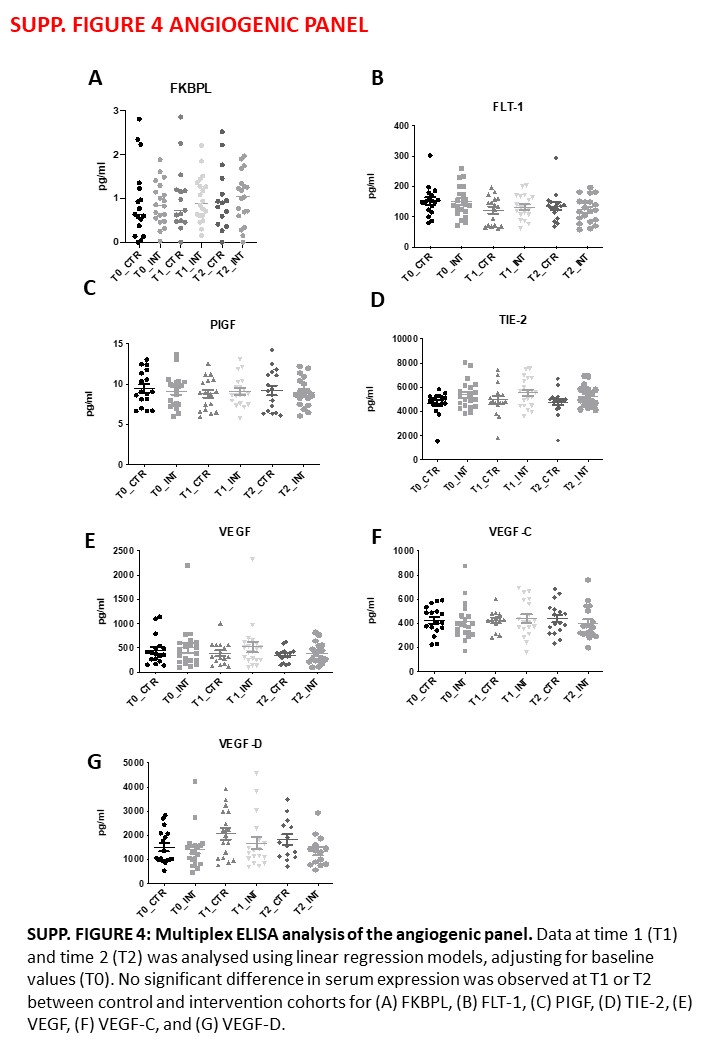

Supplement: Supplementary file 4 [file Image_4.jpeg]

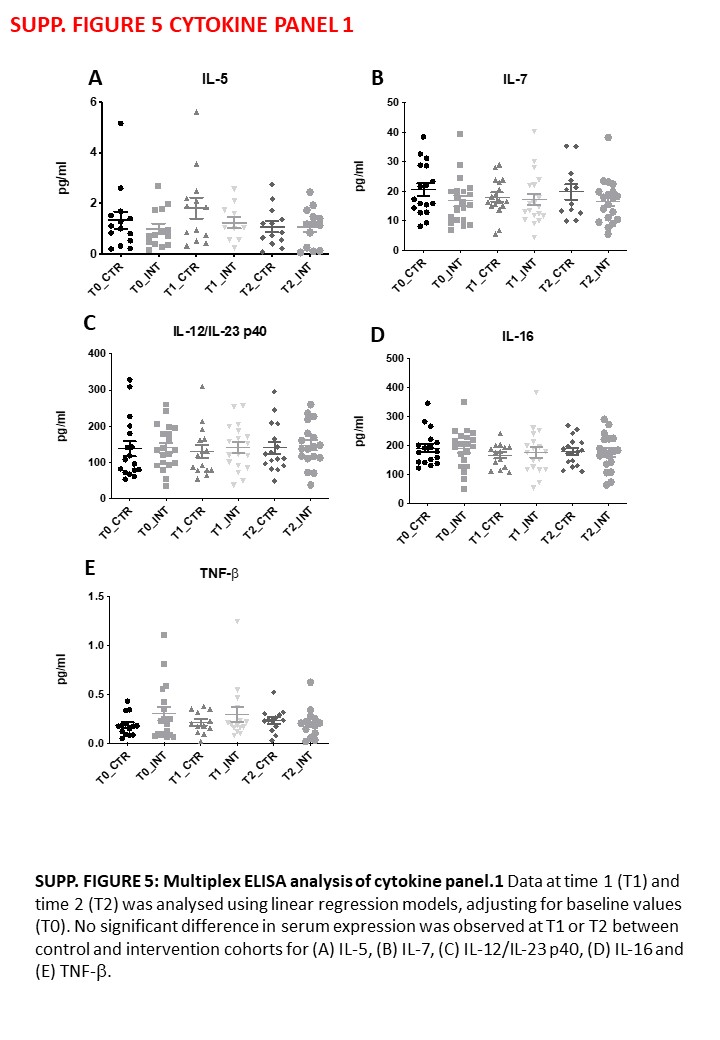

Supplement: Supplementary file 5 [file Image_5.jpeg]

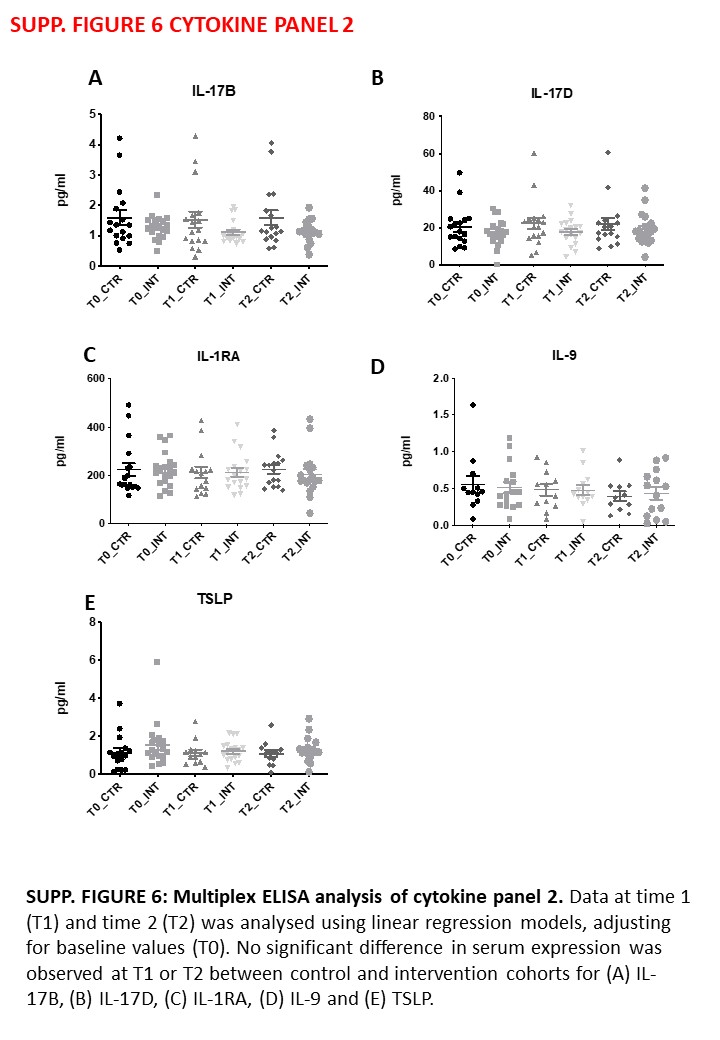

Supplement: Supplementary file 6 [file Image_6.jpeg]

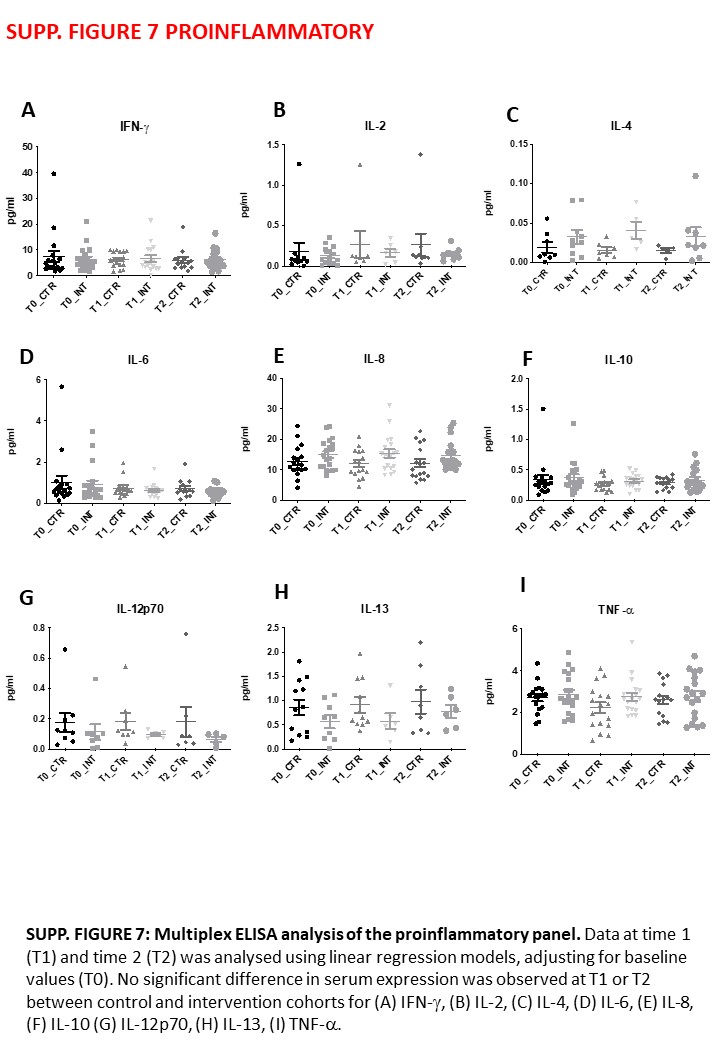

Supplement: Supplementary file 7 [file Image_7.jpeg]
