# Supplementary material for: Effect of the Rehabilitation Program, ReStOre, on Serum Biomarkers in a Randomized Control Trial of Esophagogastric Cancer Survivors
Source: Front Oncol. 2021 Sep 15;11:669078. doi: 10.3389/fonc.2021.669078 (PMC8479183; doi:10.3389/fonc.2021.669078)
Supplement: Supplementary file 8 [file Table_1.docx]

**Supplementary Material: TABLES**

**Supplementary Table 1: Correlation analysis between body mass index and serum biomarkers**

| **Biomarker** | **Spearman’s correlation coefficient (r)** | **95% Confidence Interval (CI)** | **P (two-tailed)** |
| --- | --- | --- | --- |
| Angio VEGF | 0.33 | -0.01 to 0.59 | 0.05 |
| bFGF | 0.31 | -0.03 to 0.58 | 0.06 |
| CRP | 0.30 | -0.04 to 0.58 | 0.07 |
| Eotaxin | 0.10 | -0.24 to 0.42 | 0.55 |
| Eotaxin-3 | 0.28 | -0.06 to 0.56 | 0.09 |
| Flt-1 | 0.07 | -0.27 to 0.39 | 0.68 |
| ICAM-1 | -0.09 | -0.41 to 0.25 | 0.60 |
| IFN-γ | 0.26 | -0.08 to 0.55 | 0.12 |
| IL-10 | 0.28 | -0.06 to 0.56 | 0.09 |
| **IL-12/IL-23p40** | **0.51** | **0.21 to 0.72** | **<0.01** |
| IL-12p70 | 0.11 | -0.41 to 0.57 | 0.68 |
| IL-13 | -0.21 | -0.60 to 0.25 | 0.35 |
| IL-15 | -0.01 | -0.34 to 0.33 | 0.96 |
| IL-16 | 0.148 | -0.20 to 0.45 | 0.42 |
| **IL-17A** | **0.33** | **0.002 to 0.60** | **0.04** |
| IL-17B | 0.13 | -0.21 to 0.44 | 0.44 |
| IL-17D | -0.04 | -0.37 to 0.30 | 0.81 |
| **IL-1RA** | **0.39** | **0.07 to 0.64** | **0.02** |
| IL-2 | -0.04 | -0.44 to 0.37 | 0.84 |
| IL-22 | -0.10 | -0.42 to 0.24 | 0.56 |
| IL-27 | 0.05 | -0.29 to 0.37 | 0.79 |
| IL-4 | 0.21 | -0.29 to 0.63 | 0.39 |
| IL-5 | 0.12 | -0.27 to 0.48 | 0.53 |
| IL-6 | 0.32 | -0.02 to 0.59 | 0.05 |
| IL-7 | 0.13 | -0.21 to 0.44 | 0.44 |
| IL-8 | 0.06 | -0.28 to 0.38 | 0.74 |
| IL-9 | 0.35 | -0.04 to 0.65 | 0.07 |
| IP-10 | 0.27 | -0.07 to 0.56 | 0.10 |
| MCP-1 | 0.22 | -0.12 to 0.51 | 0.20 |
| MCP-4 | 0.18 | -0.16 to 0.49 | 0.28 |
| MDC | -0.11 | -0.43 to 0.23 | 0.52 |
| MIP-1α | 0.18 | -0.18 to 0.50 | 0.30 |
| MIP-1β | 0.23 | -0.11 to 0.52 | 0.17 |
| MIP-3α | 0.07 | -0.27 to 0.40 | 0.66 |
| PlGF | 0.27 | -0.07 to 0.55 | 0.11 |
| SAA | 0.20 | -0.15 to 0.50 | 0.25 |
| TARC | -0.12 | -0.43 to 0.23 | 0.50 |
| Tie-2 | -0.05 | -0.37 to 0.29 | 0.79 |
| TNF-α | 0.23 | -0.11 to 0.52 | 0.17 |
| **TNF-β** | **0.53** | **0.21 to 0.74** | **<0.012** |
| TSLP | 0.03 | -0.31 to 0.37 | 0.86 |
| VCAM-1 | -0.05 | -0.38 to 0.29 | 0.77 |
| **VEGF** | **0.34** | **0.01 to 0.60** | **0.04** |
| VEGF-C | 0.12 | -0.23 to 0.43 | 0.50 |
| VEGF-D | -0.11 | -0.43 to 0.23 | 0.52 |
| FKBPL | -0.18 | -0.48 to 0.17 | 0.30 |

**Supplementary Table 2: Correlation analysis between 6 minute walk test and serum biomarkers**

| **Biomarker** | **Spearman’s correlation coefficient (r)** | **95% Confidence Interval (CI)** | **P (two-tailed)** |
| --- | --- | --- | --- |
| Angio VEGF | 0.24 | -0.01 to 0.53 | 0.15 |
| bFGF | 0.08 | -0.26 to 0.40 | 0.64 |
| CRP | -0.02 | -0.35 to 0.32 | 0.92 |
| Eotaxin | -0.24 | -0.53 to 0.10 | 0.16 |
| Eotaxin-3 | -0.15 | -0.46 to 0.19 | 0.37 |
| Flt-1 | -0.09 | -0.41 to 0.25 | 0.60 |
| ICAM-1 | -0.16 | -0.47 to 0.18 | 0.35 |
| IFN-γ | -0.15 | -0.46 to 0.19 | 0.37 |
| **IL-10** | **0.37** | **0.04 to 0.62** | **0.03** |
| IL-12/IL-23p40 | -0.02 | -0.35 to 0.32 | 0.93 |
| IL-12p70 | -0.13 | -0.58 to 0.39 | 0.63 |
| IL-13 | -0.34 | -0.68 to 0.13 | 0.14 |
| IL-15 | -0.03 | -0.36 to 0.30 | 0.84 |
| IL-16 | -0.10 | -0.42 to 0.24 | 0.55 |
| **IL-17A** | **-0.34** | **-0.61 to -0.01** | **0.04** |
| IL-17B | -0.29 | -0.57 to 0.05 | 0.09 |
| **IL-17D** | **-0.33** | **-0.60 to 0.01** | **0.05** |
| IL-1RA | -0.20 | -0.50 to 0.14 | 0.23 |
| IL-2 | -0.11 | -0.49 to 0.31 | 0.60 |
| IL-22 | -0.28 | -0.56 to 0.06 | 0.10 |
| IL-27 | -0.23 | -0.52 to 0.12 | 0.18 |
| IL-4 | 0.044 | -0.44 to 0.51 | 0.86 |
| IL-5 | 0.12 | -0.28 to 0.48 | 0.56 |
| IL-6 | -0.01 | -0.34 to 0.32 | 0.95 |
| IL-7 | 0.13 | -0.21 to 0.44 | 0.45 |
| IL-8 | -0.22 | -0.52 to 0.12 | 0.18 |
| IL-9 | 0.08 | -0.31 to 0.45 | 0.68 |
| IP-10 | -0.28 | -0.56 to 0.05 | 0.09 |
| MCP-1 | 0.20 | -0.14 to 0.5 | 0.24 |
| MCP-4 | 0.02 | -0.32 to 0.35 | 0.92 |
| MDC | -0.26 | -0.54 to 0.08 | 0.13 |
| MIP-1α | -0.06 | -0.40 to 0.29 | 0.72 |
| MIP-1β | -0.054 | -0.38 to 0.28 | 0.75 |
| **MIP-3α** | **-0.37** | **-0.62 to -0.04** | **0.02** |
| PlGF | 0.01 | -0.32 to 0.34 | 0.94 |
| SAA | 0.04 | -0.29 to 0.37 | 0.78 |
| TARC | -0.22 | -0.52 to 0.12 | 0.19 |
| Tie-2 | -0.22 | -0.51 to 0.13 | 0.20 |
| TNF-α | -0.14 | -0.45 to 0.20 | 0.40 |
| TNF-β | 0.12 | -0.25 to 0.46 | 0.50 |
| TSLP | 0.09 | -0.26 to 0.42 | 0.60 |
| VCAM-1 | -0.31 | -0.59 to 0.02 | 0.06 |
| VEGF | 0.19 | -0.16 to 0.49 | 0.27 |
| VEGF-C | 0.05 | -0.29 to 0.38 | 0.76 |
| VEGF-D | -0.25 | -0.54 to 0.09 | 0.13 |
| FKBPL | 0.11 | -0.24 to 0.42 | 0.53 |

**Supplementary Table 3: Correlation analysis between anaerobic threshold (AT) and serum biomarkers**

| **Biomarker** | **Spearman’s correlation coefficient (r)** | **95% Confidence Interval (CI)** | **P (two-tailed)** |
| --- | --- | --- | --- |
| Angio VEGF | -0.07 | -0.40 to 0.27 | 0.66 |
| bFGF | -0.17 | -0.47 to 0.18 | 0.33 |
| CRP | -0.05 | -0.38 to 0.29 | 0.78 |
| Eotaxin | -0.22 | -0.52 to 0.12 | 0.18 |
| Eotaxin-3 | -0.09 | -0.41 to 0.25 | 0.62 |
| Flt-1 | 0.03 | -0.30 to 0.36 | 0.85 |
| ICAM-1 | 0.13 | -0.21 to 0.44 | 0.45 |
| **IFN-γ** | **-0.34** | **-0.60 to -0.01** | **0.04** |
| IL-10 | 0.19 | -0.15 to 0.49 | 0.26 |
| IL-12/IL-23p40 | -0.31 | -0.58 to 0.029 | 0.06 |
| IL-12p70 | 0.28 | -0.25 to 0.68 | 0.28 |
| IL-13 | -0.003 | -0.45 to 0.44 | 0.99 |
| IL-15 | -0.07 | -0.40 to 0.27 | 0.67 |
| IL-16 | -0.16 | -0.47 to 0.18 | 0.34 |
| **IL-17A** | **-0.35** | **-0.61 to -0.02** | **0.03** |
| **IL-17B** | **-0.43** | **-0.67 to -0.12** | **0.01** |
| IL-17D | -0.24 | -0.53 to 0.10 | 0.14 |
| IL-1RA | -0.25 | -0.54 to 0.09 | 0.13 |
| IL-2 | 0.13 | -0.29 to 0.51 | 0.54 |
| IL-22 | -0.22 | -0.52 to 0.12 | 0.19 |
| IL-27 | -0.30 | -0.57 to 0.04 | 0.07 |
| IL-4 | 0.23 | -0.28 to 0.64 | 0.37 |
| IL-5 | 0.15 | -0.24 to 0.51 | 0.43 |
| IL-6 | -0.07 | -0.37 to 0.27 | 0.67 |
| IL-7 | -0.20 | -0.50 to 0.14 | 0.23 |
| IL-8 | -0.21 | -0.51 to 0.13 | 0.22 |
| IL-9 | -0.11 | -0.48 to 0.28 | 0.57 |
| **IP-10** | **-0.37** | **-0.62 to -0.04** | **0.02** |
| MCP-1 | -0.05 | -0.38 to 0.29 | 0.76 |
| MCP-4 | -0.11 | -0.43 to 0.23 | 0.53 |
| MDC | -0.31 | -0.58 to 0.03 | 0.07 |
| MIP-1α | 0.01 | -0.34 to 0.35 | 0.98 |
| MIP-1β | 0.03 | -0.31 to 0.36 | 0.88 |
| MIP-3α | -0.20 | -0.50 to 0.14 | 0.24 |
| PlGF | -0.28 | -0.56 to 0.06 | 0.09 |
| SAA | 0 | -0.33 to 0.33 | >0.99 |
| TARC | -0.20 | -0.50 to 0.15 | 0.24 |
| Tie-2 | -0.04 | -0.37 to 0.30 | 0.82 |
| TNF-α | -0.17 | -0.48 to 0.17 | 0.30 |
| TNF-β | 0.07 | -0.30 to 0.42 | 0.71 |
| TSLP | -0.22 | -0.52 to 0.13 | 0.21 |
| VCAM-1 | 0.03 | -0.30 to 0.36 | 0.86 |
| VEGF | -0.11 | -0.43 to 0.23 | 0.51 |
| VEGF-C | -0.11 | -0.43 to 0.23 | 0.53 |
| VEGF-D | -0.29 | -0.56 to 0.05 | 0.09 |
| FKBPL | 0.19 | -0.16 to 0.49 | 0.27 |

**Supplementary Table 4: Correlation analysis between VO_2_ max and serum biomarkers**

| **Biomarker** | **Spearman’s correlation coefficient (r)** | **95% Confidence Interval (CI)** | **P (two-tailed)** |
| --- | --- | --- | --- |
| Angio VEGF | -0.06 | -0.38 to 0.28 | 0.73 |
| bFGF | -0.22 | -0.51 to 0.12 | 0.20 |
| CRP | 0.01 | -0.32 to 0.34 | 0.95 |
| Eotaxin | -0.23 | -0.52 to 0.11 | 0.18 |
| Eotaxin-3 | -0.14 | -0.45 to 0.20 | 0.41 |
| Flt-1 | 0.02 | -0.31 to 0.35 | 0.90 |
| ICAM-1 | 0.13 | -0.21 to 0.44 | 0.44 |
| IFN-γ | -0.25 | -0.54 to 0.09 | 0.14 |
| IL-10 | 0.12 | -0.22 to 0.43 | 0.49 |
| IL-12/IL-23p40 | -0.23 | -0.52 to 0.12 | 0.18 |
| IL-12p70 | 0.23 | -0.30 to 0.65 | 0.37 |
| IL-13 | -0.01 | -0.45 to 0.43 | 0.96 |
| IL-15 | 0.08 | -0.26 to 0.40 | 0.64 |
| IL-16 | -0.12 | -0.43 to 0.22 | 0.49 |
| **IL-17A** | **-0.35** | **-0.61 to -0.02** | **0.03** |
| IL-17B | -0.22 | -0.51 to 0.12 | 0.19 |
| IL-17D | -0.14 | -0.45 to 0.21 | 0.42 |
| IL-1RA | -0.29 | -0.57 to 0.05 | 0.08 |
| IL-2 | 0.12 | -0.30 to 0.50 | 0.58 |
| IL-22 | -0.17 | -0.48 to 0.17 | 0.31 |
| IL-27 | -0.23 | -0.52 to 0.12 | 0.18 |
| IL-4 | -0.16 | -0.59 to 0.35 | 0.54 |
| IL-5 | 0.15 | -0.25 to 0.50 | 0.46 |
| IL-6 | -0.03 | -0.36 to 0.31 | 0.88 |
| IL-7 | -0.10 | -0.42 to 0.24 | 0.56 |
| IL-8 | -0.23 | -0.53 to 0.11 | 0.16 |
| IL-9 | -0.08 | -0.45 to 0.32 | 0.70 |
| IP-10 | -0.27 | -0.56 to 0.07 | 0.10 |
| MCP-1 | 0.04 | -0.30 to 0.37 | 0.81 |
| MCP-4 | -0.11 | -0.43 to 0.23 | 0.50 |
| MDC | -0.30 | -0.57 to 0.04 | 0.08 |
| MIP-1α | -0.0002 | -0.35 to 0.35 | >0.99 |
| MIP-1β | 0.10 | -0.24 to 0.42 | 0.54 |
| MIP-3α | -0.18 | -0.49 to 0.16 | 0.28 |
| PlGF | -0.15 | -0.46 to 0.19 | 0.38 |
| SAA | 0.07 | -0.27 to 0.39 | 0.68 |
| TARC | -0.23 | -0.52 to 0.12 | 0.18 |
| Tie-2 | -0.04 | -0.37 to 0.30 | 0.81 |
| TNF-α | -0.03 | -0.36 to 0.31 | 0.86 |
| TNF-β | 0.15 | -0.22 to 0.48 | 0.41 |
| TSLP | -0.13 | -0.46 to 0.22 | 0.44 |
| VCAM-1 | 0.01 | -0.32 to 0.34 | 0.95 |
| VEGF | -0.12 | -0.43 to 0.23 | 0.50 |
| VEGF-C | -0.13 | -0.44 to 0.22 | 0.45 |
| **VEGF-D** | **-0.37** | **-0.63 to -0.04** | **0.02** |
| FKBPL | -0.01 | -0.34 to 0.33 | 0.97 |

**Supplementary Table 5: Correlation analysis between fatigue score and serum biomarkers**

| **Biomarker** | **Spearman’s correlation coefficient (r)** | **95% Confidence Interval (CI)** | **P (two-tailed)** |
| --- | --- | --- | --- |
| Angio VEGF | -0.10 | -0.42 to 0.24 | 0.56 |
| bFGF | -0.04 | -0.37 to 0.29 | 0.80 |
| CRP | 0.06 | -0.28 to 0.38 | 0.74 |
| Eotaxin | -0.08 | -0.40 to 0.26 | 0.62 |
| Eotaxin-3 | -0.03 | -0.36 to 0.31 | 0.87 |
| Flt-1 | -0.17 | -0.48 to 0.17 | 0.31 |
| ICAM-1 | 0.11 | -0.24 to 0.42 | 0.53 |
| IFN-γ | 0.08 | -0.26 to 0.40 | 0.64 |
| IL-10 | 0.22 | -0.19 to 0.52 | 0.18 |
| IL-12/IL-23p40 | -0.14 | -0.45 to 0.21 | 0.42 |
| IL-12p70 | -0.23 | -0.65 to 0.30 | 0.37 |
| IL-13 | 0.21 | -0.25 to 0.60 | 0.36 |
| IL-15 | -0.09 | -0.41 to 0.25 | 0.58 |
| IL-16 | -0.23 | -0.52 to 0.11 | 0.17 |
| IL-17A | 0.09 | -0.25 to 0.41 | 0.58 |
| IL-17B | -0.02 | -0.35 to 0.31 | 0.90 |
| IL-17D | 0.15 | -0.19 to 0.46 | 0.36 |
| IL-1RA | 0.02 | -0.32 to 0.35 | 0.91 |
| IL-2 | 0.01 | -0.40 to 0.42 | 0.95 |
| IL-22 | 0.22 | -0.12 to 0.52 | 0.19 |
| IL-27 | 0.16 | -0.18 to 0.47 | 0.35 |
| IL-4 | 0.12 | -0.38 to 0.57 | 0.62 |
| IL-5 | -0.36 | -0.66 to 0.02 | 0.06 |
| IL-6 | 0.18 | -0.17 to 0.48 | 0.30 |
| IL-7 | -0.26 | -0.54 to 0.08 | 0.12 |
| IL-8 | 0.08 | -0.26 to 0.40 | 0.64 |
| IL-9 | -0.17 | -0.52 to 0.23 | 0.40 |
| IP-10 | 0.21 | -0.13 to 0.51 | 0.20 |
| MCP-1 | -0.12 | -0.44 to 0.22 | 0.48 |
| MCP-4 | -0.10 | -0.42 to 0.24 | 0.55 |
| MDC | 0.02 | -0.31 to 0.35 | 0.89 |
| MIP-1α | 0.32 | -0.04 to 0.60 | 0.07 |
| MIP-1β | -0.05 | -0.38 to 0.28 | 0.75 |
| MIP-3α | -0.10 | -0.42 to 0.24 | 0.55 |
| PlGF | -0.09 | -0.41 to 0.25 | 0.61 |
| SAA | 0.002 | -0.33 to 0.33 | 0.99 |
| TARC | -0.03 | -0.36 to 0.30 | 0.85 |
| Tie-2 | -0.09 | -0.41 to 0.25 | 0.59 |
| TNF-α | -0.12 | -0.43 to 0.22 | 0.49 |
| TNF-β | 0.02 | -0.34 to 0.38 | 0.91 |
| TSLP | -0.24 | -0.54 to 0.11 | 0.17 |
| VCAM-1 | -0.04 | -0.37 to 0.30 | 0.81 |
| VEGF | -0.03 | -0.36 to 0.30 | 0.84 |
| VEGF-C | -0.13 | -0.44 to 0.21 | 0.44 |
| VEGF-D | 0.08 | -0.26 to 0.40 | 0.64 |
| FKBPL | 0.14 | -0.22 to 0.45 | 0.40 |

**Supplementary Table 6: Changes in serum biomarkers from pre to post intervention**

| **Biomarker** | **Group** | **n** | **Baseline (T0)**  **mean (SD)** | **n** | **Immediate follow up (T1)**  **mean (SD)** | **Mean difference***  **(95% confidence interval)**  **p-value** | **n** | **3 month follow up (T2)**  **mean (SD)** | **Mean difference***  **(95% confidence interval)**  **p-value** |
| --- | --- | --- | --- | --- | --- | --- | --- | --- | --- |
| **Vascular Injury Panel** | | | | | | | | | |
| CRP | control | 17 | 6219829 (15900000) | 13 | 1412965 (1152707) | 0.76 (0.22 to 2.63)  0.66 | 13 | 1749907 (1937039) | 1.12 (0.22 to 5.75)  0.89 |
|  | intervention | 20 | 2207638  (2445631) | 15 | 1510592 (1291289) |  | 16 | 1626784 (1579491) |  |
| ICAM1 | control | 17 | 438206.6 (156874.3) | 17 | 383271.8 (145317.2) | **1.05 (1.07 to 1.66)**  **0.02** | 17 | 431376  (166098.2) | 1.05 (0.63 to 1.78)  0.84 |
|  | intervention | 20 | 438392.7 (181872.8) | 20 | 469362.7 (144530.5) |  | 20 | 424143.9 (124105.8) |  |
| SAA | control | 17 | 20000000 (7.15E+07) | 14 | 2211782 (1307138) | 1.54 (0.63 to 3.8)  0.32 | 13 | 2347886 (1331342) | 1.44 (0.41 to 5.13)  0.56 |
|  | intervention | 20 | 2582092  (2456469) | 17 | 2872279 (1780062) |  | 16 | 2515205 (1932047) |  |
| VCAM1 | control | 17 | 730543  (281855.3) | 17 | 671478.8 (262585.7) | **1.51 (1.04 to 2.14)**  **0.02** | 16 | 727438.2 (311298.1) | 0.98 (0.60 to 1,62)  0.94 |
|  | intervention | 20 | 734501.4 (258487.9) | 19 | 818073.3 (268972.8) |  | 19 | 735680.7 (248094.7) |  |
| **Proinflammatory Panel** | | | | | | | | | |
| IFN-γ | control | 17 | 7.57  (9.24) | 16 | 6.33  (3.06) | 0.21 (-2.75 to 3.18)  0.88 | 14 | 6.02  (4.21) | -0.23 (-1.96 to 1.50)  0.79 |
|  | intervention | 20 | 6.45  (4.51) | 16 | 6.59  (5.43) |  | 16 | 6.31  (3.66) |  |
| IL-2 | control | 11 | 0.18  (0.36) | 7 | 0.26  (0.44) | -0.02 (-0.10 to 0.05)  0.52 | 9 | 0.26  (0.43) | -0.01 (-0.07 to 0.06)  0.86 |
|  | intervention | 14 | 0.13  (0.11) | 6 | 0.17  (0.12) |  | 8 | 0.15  (0.08) |  |
| IL-4 | control | 8 | 0.02  (0.02) | 7 | 0.02  (0.01) | 0.02 (0 to 0.04)  0.06 | 6 | 0.02  (0.01) | 0.01 (-0.02 to 0.04)  0.61 |
|  | intervention | 10 | 0.03  (0.03) | 5 | 0.04  (0.02) |  | 8 | 0.03  (0.03) |  |
| IL-6 | control | 17 | 1.03  (1.31) | 14 | 0.75  (0.46) | -0.05 (-0.19 to 0.10)  0.49 | 13 | 0.73  (0.45) | -0.04 (-0.23 to 0.14)  0.64 |
|  | intervention | 20 | 0.92  (0.87) | 18 | 0.63  (0.31) |  | 17 | 0.57  (0.24) |  |
| IL-8 | control | 17 | 12.83 (5.1) | 16 | 12.12 (4.55) | 0.73 (-1.54 to 3.00)  0.52 | 17 | 12.22 (5.48) | 1.49 (-1.03 to 4.02) 0.24 |
|  | intervention | 20 | 15  (4.85) | 19 | 15.4  (5.9) |  | 18 | 14.83 (4.72) |  |
| IL 10 | control | 17 | 0.34  (0.32) | 16 | 0.27  (0.11) | 0.02 (-0.04 to 0.08)  0.42 | 15 | 0.28  (0.09) | 0.01 (-0.08 to 0.10)  0.81 |
|  | intervention | 20 | 0.37  (0.26) | 17 | 0.31  (0.12) |  | 17 | 0.33  (0.18) |  |
| IL-12p70 | control | 9 | 0.18  (0.19) | 8 | 0.18  (0.16) | -0.01 (-0.07 to 0.06)  0.75 | 7 | 0.18  (0.26) | 0.01 (-0.05 to 0.07)  0.66 |
|  | intervention | 8 | 0.11  (0.15) | 5 | 0.10  (0.02) |  | 5 | 0.06  (0.04) |  |
| IL-13 | control | 12 | 0.86  (0.56) | 11 | 0.91  (0.52) | -0.15 (-0.54 to 0.24)  0.43 | 8 | 0.98  (0.70) | 0.01 (-0.37 to 0.40)  0.95 |
|  | intervention | 9 | 0.57  (0.40) | 6 | 0.58  (0.40) |  | 6 | 0.78  (0.34) |  |
| TNF-α | control | 17 | 2.71  (0.72) | 17 | 2.23  (1.04) | 0.34 (-0.22 to 0.91)  0.23 | 15 | 2.59  (0.81) | 0.07 (-0.29 to 0.44)  0.68 |
|  | intervention | 20 | 2.87  (0.97) | 19 | 2.75  (0.84) |  | 18 | 2.76  (1.08) |  |
| **Chemokine Panel** | | | | | | | | | |
| Eotaxin | control | 17 | 361.51 (136.99) | 14 | 356.07 (175.74) | -7.72 (-52.27 to 36.84)  0.73 | 17 | 331.82 (122.06) | 8.81 (-55.87 to 73.48)  0.78 |
|  | intervention | 20 | 444.45  (291) | 17 | 421.53 (294.62) |  | 19 | 403.78 (305.26) |  |
| Eotaxin-3 | control | 17 | 11.42  (8.28) | 16 | 8.17  (5.07) | 1.11 (0.69 to 1.78)*  0.66 | 15 | 6.87  (5.13) | **2.59 (0.23 to 4.96) 0.03** |
|  | intervention | 19 | 11  (9.9) | 16 | 9.56  (6.31) |  | 18 | 11.36  (9.82) |  |
| IP-10 | control | 17 | 195.99  (86.77) | 16 | 144.87  (52.6) | **38.02 (0.69 to 75.35)**  **0.05** | 14 | 155.97 (72.17) | 12.25 (-23.37 to 47.78)  0.49 |
|  | intervention | 19 | 201.5  (71.2) | 18 | 192.17  (79.87) |  | 17 | 179.82 (62.14) |  |
| MCP-1 | control | 17 | 283.99  (71.34) | 16 | 281  (55.87) | 3.05 (-26.65 to 32.76)  0.84 | 17 | 277.1  (70.07) | 16.35 (-24.52 to 57.23)  0.42 |
|  | intervention | 20 | 315.19 (172.08) | 20 | 313.33 (159.68) |  | 20 | 321.52 (172.81) |  |
| MCP-4 | control | 17 | 171.55  (78.06) | 15 | 191.97  (84.81) | -21.26 (-46.9 to 4.37)  0.10 | 15 | 177.22 (87.27) | -4.81 (-26.13 to 16.51)  0.65 |
|  | intervention | 20 | 182.93  (97.98) | 19 | 177.6  (69.4) |  | 20 | 192.12 (84.15) |  |
| MIP1-α | control | 15 | 13.06  (5.23) | 13 | 14.07  (3.95) | 0.44 (-3.73 to 2.85)  0.79 | 15 | 14.28  (6.36) | -1.31 (-5.16 to 2.54)  0.49 |
|  | intervention | 19 | 16.9 (9.77) | 17 | 16.13  (8.17) |  | 16 | 16.98  (8.55) |  |
| MDC | control | 17 | 1391.56 (430.33) | 17 | 1350.38 (393.86) | 0.24 (-102.83 to 103.31)  0.99 | 17 | 1346.19 (444.27) | -61.02 (-174.25 to 52.21)  0.28 |
|  | intervention | 20 | 1948.69 (2114.65) | 18 | 1424.45 (410.15) |  | 19 | 1365.09 (379.96) |  |
| MIP1-β | control | 17 | 113.3  (50.46) | 17 | 109.4  (46.62) | 6.9 (-3.69 to 17.49)  0.19 | 16 | 113.62 (61.79) | 1.05 (0.81 to 1.35)  0.70 |
|  | intervention | 20 | 106.38  (39.03) | 20 | 109.74  (43.65) |  | 19 | 107.35 (41.27) |  |
| TARC | control | 17 | 433.01 (279.68) | 15 | 418.86 (264.19) | 18.88 (-31.42 to 69.17)  0.45 | 13 | 320.43 (234.39) | 35.18 (-8.85 to 79.21)  0.11 |
|  | intervention | 20 | 400.01  (305.9) | 18 | 403.03  (310.4) |  | 17 | 373.39 (302.36) |  |
| **TH17 Panel** | | | | | | | | | |
| IL-17A | control | 17 | 2.71  (2.99) | 16 | 2.04  (1.66) | 0.66 (-0.11 to 1.42)  0.09 | 16 | 2.66  (1.85) | -0.42 (-1.07 to 0.24)  0.20 |
|  | intervention | 20 | 2.05  (2.24) | 17 | 2.2  (1.47) |  | 18 | 1.71  (1) |  |
| IL-22 | control | 16 | 1.78  (1.59) | 12 | 1.35  (1.07) | 0.17 (-0.36 to 0.69) 0.52 | 11 | 1.58  (1.23) | 0.2 (-0.47 to 0.86)  0.55 |
|  | intervention | 20 | 1.44  (1.22) | 17 | 1.49  (0.81) |  | 17 | 1.68  (1.44) |  |
| IL-27 | control | 17 | 2006.95 (920.82) | 16 | 1760.85 (763.50) | **249.48 (22.43 to 476.53)**  **0.03** | 16 | 1816.47 (741.08) | 17.74 (-215.23 to 250.71)  0.88 |
|  | intervention | 20 | 2106.86 (633.06) | 19 | 2198.45 (643.23) |  | 19 | 1969.87 (535.45) |  |
| MIP3-α | control | 17 | 8.62  (7.21) | 16 | 7.67  (4.17) | 0.33 (-1.71 to 2.38)  0.74 | 16 | 9.64  (6.84) | -0.7 (-2.57 to 1.16)  0.45 |
|  | intervention | 20 | 20.12 (47.36) | 17 | 9.94  (13.92) |  | 16 | 5.84  (5.04) |  |
| **Angiogenic Panel** | | | | | | | | | |
| Angio-VEGF | control | 17 | 454.43 (299.84) | 16 | 390.63  (232.68) | 44.95 (-25.78 to 115.68) 0.20 | 14 | 352.34 (150.58) | 4.35 (-37.54 to 46.25)  0.83 |
|  | intervention | 20 | 500.44 (455.16) | 20 | 521.18  (478.75) |  | 18 | 389.98 (214.81) |  |
| bFGF | control | 17 | 5.66  (6.58) | 14 | 4.74  (3.19) | -1.33 (-2.81 to 0.15)  0.08 | 14 | 4.11  (2.89) | **-1.62 (-2.99 to -0.26)**  **0.02** |
|  | intervention | 20 | 6.38  (4.49) | 18 | 5.05  (3.5) |  | 16 | 3.07  (2.25) |  |
| Flt-1 | control | 17 | 152.36 (51.01) | 17 | 121.48  (46.35) | 13.7 (-9.8 to 37.21)  0.24 | 16 | 134.6  (51.35) | -8.1 (-28.96 to 12.75)  0.43 |
|  | intervention | 20 | 150.97 (52.98) | 19 | 132.23  (40.64) |  | 20 | 122.7  (42.7) |  |
| PIGF | control | 17 | 9.48  (2.18) | 17 | 8.81  (2.02) | 0.7 (-0.07 to 1.48)  0.07 | 17 | 9.23  (2.51) | -0.09 (-1.03 to 0.86)  0.85 |
|  | intervention | 20 | 9.16  (2.05) | 19 | 9.1  (1.85) |  | 19 | 9.01  (1.79) |  |
| VEGF | control | 17 | 89.60 (58.95) | 14 | 77.96  (40.37) | 12.46 (-6.41 to 31.33)  0.19 | 15 | 83.77  (62.85) | 1.6 (-13.31 to 16.52)  0.83 |
|  | intervention | 20 | 98.03 (124.49) | 18 | 84.73  (50.66) |  | 17 | 71.57  (42.25) |  |
| VEGF-C | control | 17 | 426.55 (116.12) | 13 | 424.51  (87.11) | 23.71 (-54.13 to 101.55) 0.54 | 17 | 440.42 (131.19) | -1.21 (-70.6 to 68.18)  0.97 |
|  | intervention | 20 | 413. 14 (154.48) | 19 | 440.38  (150.87) |  | 18 | 402.26  (136.5) |  |
| VEGF-D | control | 17 | 1512.65 (710.25) | 17 | 2063.66 (998.58) | 0.83 (0.58 to 1.20)*  0.32 | 14 | 1825.03 (835.72) | 0.92 (0.71 to 1.19)*  0.49 |
|  | intervention | 20 | 1429.15 (826.64) | 18 | 1680.74 (1084.59) |  | 14 | 1355.97 (634.33) |  |
| FKBPL | control | 17 | 0.92  (0.84) | 15 | 0.99  (0.76) | -0.11 (-0.27 to 0.04)  0.15 | 15 | 1.00  (0.72) | 0 (-0.19 to 0.19)  0.99 |
|  | intervention | 20 | 0.88  (0.49) | 20 | 0.98  (0.53) |  | 19 | 1.01  (0.59) |  |
| TIE -2 | control | 17 | 4692.2 (959.5) | 16 | 4973.0  (1334.9) | -128.8 (-696.85 to 439.24)  0.64 | 17 | 4773.7 (1067.6) | 33.86 (-333.75 to 401.48)  0.73 |
|  | intervention | 20 | 5384.0 (1182.3) | 20 | 5543.1  (1188.8) |  | 19 | 5257.9  (852.2) |  |
| **Cytokine Panel 1** | | | | | | | | | |
| IL-5 | control | 14 | 1.33 (1.28) | 13 | 1.81  (1.5) | -0.23 (-0.68 to 0.22)  0.29 | 13 | 1.07  (0.79) | 0.95 (0.44 to 2.06)*  0.90 |
|  | intervention | 14 | 0.99 (0.76) | 10 | 1.24  (0.74) |  | 13 | 1.07  (0.76) |  |
| IL-7 | control | 17 | 20.54 (8.78) | 17 | 18.12  (6.39) | 1.83 (-1.42 to 5.08)  0.26 | 12 | 19.81  (8.87) | -0.42 (-2.78 to 1.93)  0.71 |
|  | intervention | 20 | 16.77 (7.93) | 20 | 17.24  (8.45) |  | 19 | 16.66  (7.51) |  |
| IL-12/IL-23p40 | control | 17 | 138.32 (84.47) | 15 | 131.06 (68.89) | -8.43 (-19.04 to 2.19) 0.12 | 16 | 140.3837 (67.31) | -4.2 (-21.68 to 13.29)  0.63 |
|  | intervention | 20 | 141.14 (60.47) | 18 | 141.12 (64.02) |  | 18 | 147.68 (60.23) |  |
| IL-15 | control | 17 | 3.49 (0.86) | 17 | 3.35  (0.9) | 0.21 (-0.08 to 0.50)  0.15 | 16 | 3.32  (0.81) | **0.27 (0 to 0.54)**  **0.05** |
|  | intervention | 20 | 3.4  (0.9) | 18 | 3.39  (0.84) |  | 18 | 3.49  (0.84) |  |
| IL-16 | control | 17 | 192.38 (60.41) | 14 | 167.79 (40.99) | -1.89 (-17.32 to 13.54) 0.80 | 17 | 180.5  (47.88) | 1.47 (-15.14 to 18.09) 0.86 |
|  | intervention | 20 | 190.39 (68.39) | 18 | 177.59  (77.4) |  | 18 | 176.78 (63.76) |  |
| TNF-β | control | 14 | 0.18 (0.11) | 12 | 0.21  (0.11) | 0.01 (-0.12 to 0.14)  0.85 | 13 | 0.23  (0.12) | -0.04 (-0.11 to 0.03)  0.24 |
|  | intervention | 18 | 0.30 (0.29) | 15 | 0.29  (0.29) |  | 14 | 0.2  (0.15) |  |
| **Cytokine Panel 2** | | | | | | | | | |
| IL-17B | control | 17 | 1.60 (1.01) | 17 | 1.53  (1.1) | -0.12 (-0.43 to 0.19) 0.44 | 17 | 1.58  (1.02) | -0.08 (-0.27 to 0.12)  0.43 |
|  | intervention | 20 | 1.28 (0.39) | 19 | 1.11  (0.38) |  | 18 | 1.14  (0.36) |  |
| IL-17D | control | 17 | 20.38 (10.68) | 17 | 22.21  (13.12) | -0.76 (-4.23 to 2.70) 0.66 | 16 | 21.74  (12.98) | 0.91 (-2.56 to 4.37)  0.60 |
|  | intervention | 20 | 17.41 (7.14) | 18 | 17.59  (6.98) |  | 20 | 19.22  (8.3) |  |
| IL-1RA | control | 17 | 224.54 (110.72) | 15 | 211.99 (92.74) | -10.1 (-38.72 to 18.52) 0.48 | 16 | 226.02  (73.54) | -29.96 (-69.46 to 9.55) 0.13 |
|  | intervention | 20 | 224.62 (72.49) | 18 | 212.84 (77.68) |  | 18 | 204.12  (90.73) |  |
| IL-9 | control | 12 | 0.56 (0.39) | 12 | 0.48  (0.27) | 1.01 (0.57 to 1.81)* 0.97 | 11 | 0.39  (0.22) | -0.01 (-0.2 to 0.18)  0.93 |
|  | intervention | 16 | 0.51 (0.31) | 13 | 0.48  (0.25) |  | 14 | 0.43  (0.31) |  |
| TSLP | control | 16 | 1.13 (0.93) | 13 | 1.11  (0.64) | 0.06 (-0.24 to 0.36) 0.69 | 13 | 1.07  (0.62) | 0.01 (-0.57 to 0.59)  0.97 |
|  | intervention | 19 | 1.55 (1.20) | 18 | 1.19  (0.54) |  | 17 | 1.29  (0.65) |  |

***Outliers removed and variables were logged transformed. The results presented here have been back transformed and represent the ratio of the means of the control and intervention group. Note that if there was no difference between the groups the expected value of the ratio would be 1.**
